# A Simple and Versatile Strategy for Oriented Immobilization of His-Tagged Proteins on Magnetic Nanoparticles

**DOI:** 10.1021/acs.bioconjchem.3c00417

**Published:** 2023-10-26

**Authors:** Christian Castro-Hinojosa, Susel Del Sol-Fernández, Eduardo Moreno-Antolín, Beatriz Martín-Gracia, Jesús G. Ovejero, Jesús Martínez de la Fuente, Valeria Grazú, Raluca M. Fratila, María Moros

**Affiliations:** † Instituto de Nanociencia y Materiales de Aragón, INMA (CSIC-Universidad de Zaragoza), C/Pedro Cerbuna 12, Zaragoza 50009, Spain; ‡ Instituto de Ciencia de Materiales de Madrid (ICMM/CSIC), Sor Juana Inés de la Cruz 3, Madrid 28049, Spain; § Department of Dosimetry and Radioprotection, General University Hospital Gregorio Marañón, Dr Esquerdo 46, Madrid 28007, Spain; ∥ Centro de Investigación Biomédica en Red de Bioingeniería, Biomateriales y Nanomedicina (CIBER-BBN), Madrid 28029, Spain; ⊥ Departamento de Química Orgánica, Facultad de Ciencias, Universidad de Zaragoza, C/Pedro Cerbuna 12, Zaragoza 50009, Spain

## Abstract

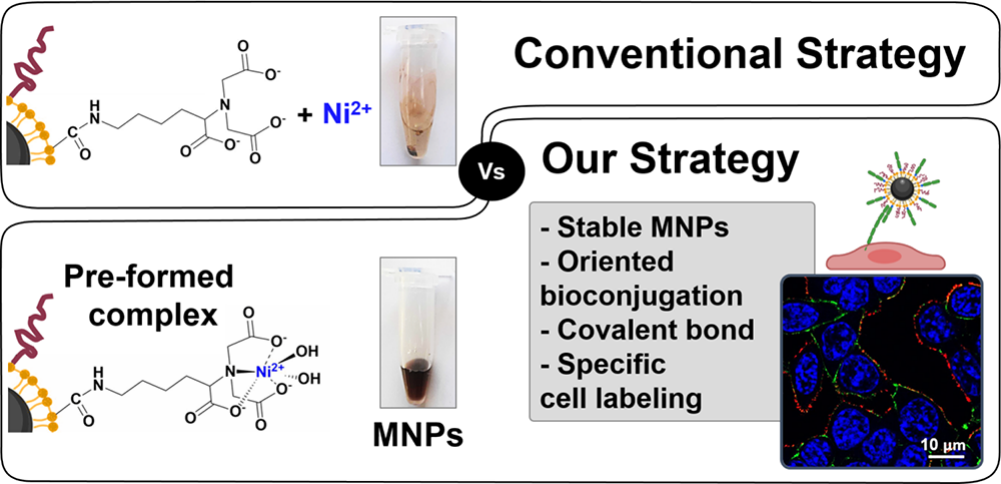

Oriented and covalent immobilization of proteins on magnetic nanoparticles (MNPs) is particularly challenging as it requires both the functionality of the protein and the colloidal stability of the MNPs to be preserved. Here, we describe a simple, straightforward, and efficient strategy for MNP functionalization with proteins using metal affinity binding. Our method involves a single-step process where MNPs are functionalized using a preformed, ready-to-use nitrilotriacetic acid-divalent metal cation (NTA-M^2+^) complex and polyethylene glycol (PEG) molecules. As a proof-of-concept, we demonstrate the oriented immobilization of a recombinant cadherin fragment engineered with a hexahistidine tag (6His-tag) onto the MNPs. Our developed methodology is simple and direct, enabling the oriented bioconjugation of His-tagged cadherins to MNPs while preserving protein functionality and the colloidal stability of the MNPs, and could be extended to other proteins expressing a polyhistidine tag. When compared to the traditional method where NTA is first conjugated to the MNPs and afterward free metal ions are added to form the complex, this novel strategy results in a higher functionalization efficiency while avoiding MNP aggregation. Additionally, our method allows for covalent bonding of the cadherin fragments to the MNP surface while preserving functionality, making it highly versatile. Finally, our strategy not only ensures the correct orientation of the protein fragments on the MNPs but also allows for the precise control of their density. This feature enables the selective targeting of E-cadherin-expressing cells only when MNPs are decorated with a high density of cadherin fragments.

## Introduction

In recent years, nanomaterials have shown enormous potential in different fields, such as biomedicine. Among other advantages, they have a high surface-to-volume ratio, they show optical and magnetic properties different to those of the bulk material and they can be functionalized with a great variety of molecules.^1^ Additionally, their size is comparable to those of most biomolecules, including cell surface receptors,^2^ thus enhancing the interaction. Magnetic nanoparticles (MNPs) have unique physicochemical properties, which allow them to respond to magnetic fields and deliver physical stimuli, such as heat or tractional forces.^3,4^ These attributes have converted MNPs into an increasingly important tool for biomedical applications such as bioseparation, magnetic resonance imaging, magnetic hyperthermia, targeted drug delivery, cell labeling, and the remote stimulation of cell receptors.^3,5−7^

For most of these applications, specific targeting of the MNPs to the desired cells is of utmost importance.^2,8^ To endow the MNPs with specific targeting capabilities, their surface is usually functionalized with antibodies, proteins, or peptides that selectively recognize cellular receptors.^3,4,9,10^ Critical factors that can ultimately affect cellular recognition are the orientation and density of the biomolecule on the MNP surface.^11^ For instance, biomolecule orientation is crucial when using antibodies, as the antigen binding site must not be involved in the functionalization process. Similarly, cell adhesion proteins such as cadherins would need to be properly oriented on the MNP surface in order to ensure specific recognition by their cellular counterparts.^12,13^ The correct biomolecule orientation is especially important when using nanoparticles as the number of biomolecules that can be functionalized on their surface is rather low. On the contrary, larger particles such as microbeads can be functionalized with many biomolecules, and thus the probability of correct interactions with the receptors is higher, even at a low coverage density or when not all the biomolecules have the correct orientation on the particle surface.^2,9,14^ Ultimately, the density and orientation of the biomolecules can affect the potential cellular responses, including the MNP cellular uptake, the intracellular trafficking, and the underlying kinetics of these interactions.^15−17^

It is therefore clear that an appropriate bioconjugation chemistry, able to control biomolecule orientation and density, could help to make specific targeting less cumbersome. Several strategies have been implemented to achieve oriented protein bioconjugation onto MNPs for cell labeling. Among them, noncovalent strategies such as the biotin–avidin interaction have been largely exploited. However, the necessity of a prior protein modification with biotin, which is often carried out using nonselective biotinylation reagents, can limit the control over the orientation.^18^ An alternative noncovalent strategy involves the bioconjugation of proteins engineered with fusion tags such as a polyhistidine tail (His-tag) to magnetic particles via metal affinity binding. In this approach, the particles are functionalized with nitrilotriacetic acid (NTA) or its derivatives and subsequently complexed with divalent metal cations (M^2+^) to generate NTA-M^2+^ complexes on the particle surface. These complexes can further coordinate in an oriented way virtually any engineered protein containing a His-tag. Due to the specificity and the stability of the union, this metal affinity binding has been largely exploited as a tool for protein separation in complex media, using magnetic microparticles or to a lesser extent nanoparticles.^19−24^ However, its use as a MNP bioconjugation strategy for cellular targeting has been barely explored.^25,26^ A possible reason is the need for several functionalization steps, first with NTA and afterward with the metal ions to form the NTA-M^2+^ complex on the MNP surface,^25,27^ resulting in time-consuming protocols. Moreover, the stability of the MNPs might be compromised due to the addition of metal ions.^21^

Here, we provide a straightforward version of this strategy in which the MNPs can be functionalized in a single step with a previously formed, ready-to-use NTA-M^2+^ complex and polyethylene glycol (PEG) molecules. This results in a faster and modulable functionalization procedure, without compromising the MNP stability. As a proof of concept for subsequent protein immobilization on the MNP surface, we have focused on cadherins as model proteins since they require a proper orientation to maintain homophilic interaction with cellular cadherins. Specifically, we have chosen an attractive cellular target, E-cadherin, which is a calcium-dependent cell adhesion protein mostly found in epithelial tissues and involved in important cellular processes, such as cell morphogenesis, embryonic development, or tissue growth.^13^ To specifically recognize E-cadherin on cellular membranes, we immobilized on the MNPs a recombinant cadherin fragment engineered with a hexahistidine tag (6His-tag) using metal affinity binding.

Our developed methodology is simple and straightforward, allowing the oriented bioconjugation of His-tagged cadherins to MNPs without compromising the protein functionality or the colloidal stability of the MNPs. Furthermore, covalent bonding of the fragments to the MNP surface is also possible, making this method highly versatile. Of note, our strategy not only ensures a correct orientation of the protein fragments on the MNPs, but also allows controlling their density. This ultimately allows for a selective targeting of E-cadherin-expressing cells only when MNPs are decorated with a high density of cadherin fragments. Given that the His-tag is one of the most widely used tags for the purification of recombinant proteins, this strategy has the potential to facilitate the bioconjugation of a wide variety of biomolecules to MNPs for cellular targeting in a fast and oriented manner.

## Results and Discussion

### Production of E-cadherin Fragments E/EC12

E-cadherins interact with other cell surface cadherins by homophilic interactions through their extracellular subdomain units (E/EC15). The minimum fragment necessary to establish these interactions consists of the two outermost cadherin modules (from now afterward referred to as E/EC12).^28^ Recombinant E/EC12 fragments were expressed in *E. coli* BL21 transformed with a plasmid bearing the protein sequence fused to a C-terminal 6His-tag as shown in Figure S1a (Supporting Information). The proper folding of the protein after purification was verified by a trypsin cleavage assay, as E-cadherin is susceptible to cleavage by proteases such as trypsin in the absence of calcium ions; in contrast, in the presence of calcium ions, a conformational change in its structure buries the cleavage sites, preventing the trypsin from reaching them.^28^ After E/EC12 was incubated with trypsin in the presence or absence of Ca^2+^, the cleavage was evaluated by SDS-PAGE electrophoresis. As shown in Figure S1b (Supporting Information), in the absence of Ca^2+^, degradation is demonstrated by the presence of additional bands with lower molecular weight; however, in the presence of Ca^2+^, the protein remained as a single band, indicating proper folding of the protein fragments.

The E/EC12 fragments were further analyzed by circular dichroism (CD), a sensitive method for evaluating the secondary structure, folding, and binding properties of proteins. The CD spectra exhibited a minimum at around 216 nm (SI, Figure S1c), typical for a β-sheet secondary structure and similar to the spectra reported for correctly folded E/EC12.^28,29^ When calcium was added, the mean residue molar ellipticity decreased, a response associated with a conformational change in the protein in the presence of calcium,^29−31^ supporting the correct folding of the protein indicated by the trypsin cleavage results.

### Synthesis and Characterization of MNPs

**Figure 1:**
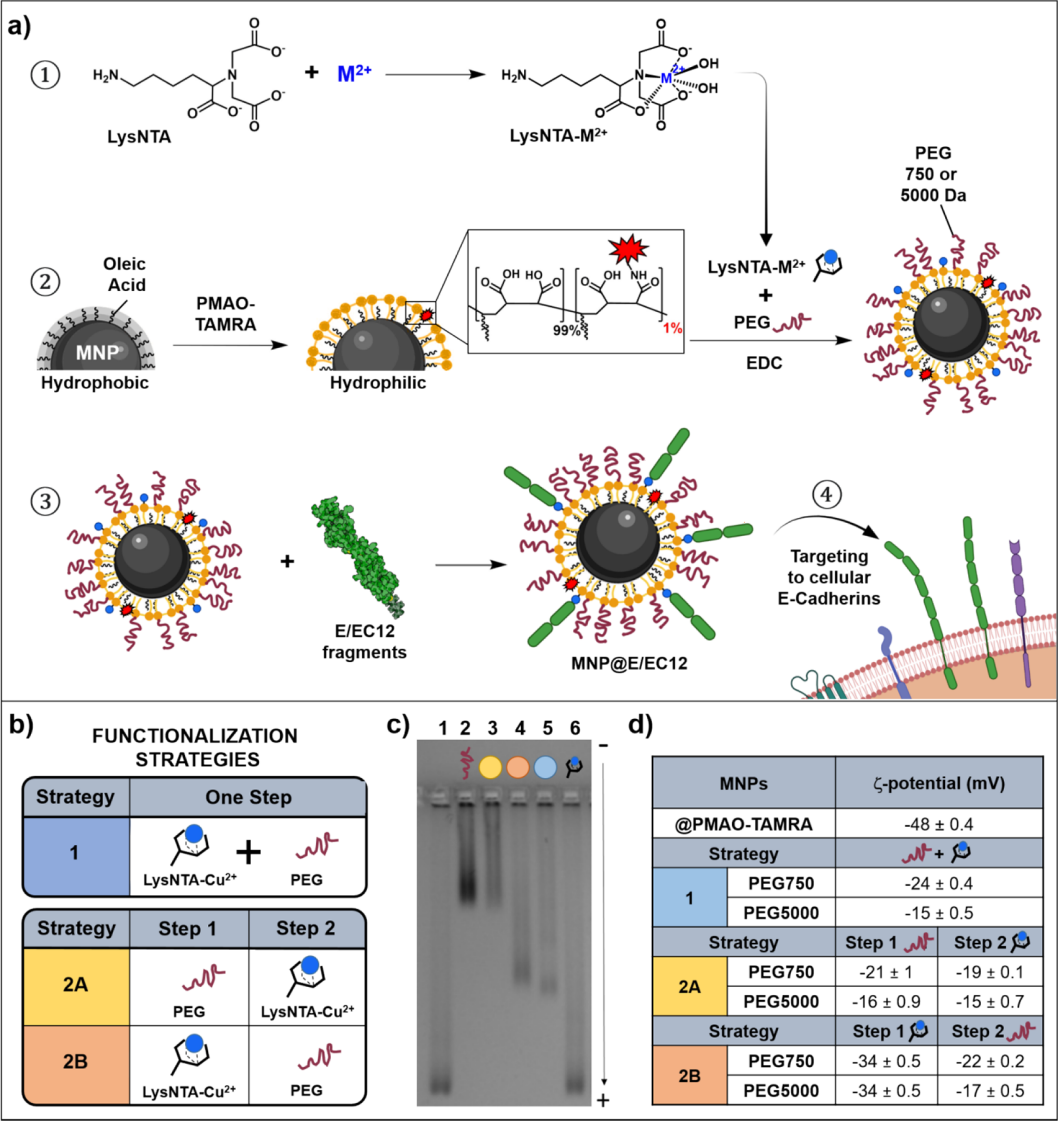
(a) Scheme of MNP bioconjugation with E/EC12 fragments. In a first step, the ready-to-use LysNTA-M^2+^ complex is formed. Thereafter, this complex and PEG molecules are functionalized on the MNPs@PMAO-TAMRA that have been activated with EDC. Finally, E/EC12 fragments are coupled to these MNPs via metal affinity binding. Figure created with BioRender.com. (b) One step and two-step functionalization strategies of the MNPs with PEG (750 or 5000 Da) and LysNTA-Cu^2+^ complexes. (c) Agarose gel electrophoresis of the MNPs functionalized with strategies 1 and 2 using PEG 750 Da. Lanes: 1: MNPs@PMAO-TAMRA; 2: MNPs@PEG-750; 3: Strategy 2A; 4: Strategy 2B; 5: Strategy 1; 6: MNPs@LysNTA-Cu^2+^. (d) ζ-potential of the MNPs obtained after each step of both functionalization strategies.

Manganese–iron oxide MNPs (Mn_0.14_Fe_2.86_O_4_) with a diameter of 14.2 ± 2.6 nm (determined from transmission electron microscopy images, TEM) were synthesized by thermal decomposition (SI, Figure S2). This method yields hydrophobic MNPs stabilized with oleic acid ligands that are soluble in organic solvents (hexane). To use the MNPs in biological media, the MNPs were transferred to the aqueous phase by coating them with an amphiphilic polymer, poly(maleic anhydride-*alt*-1-octadecene) (PMAO), which also provides carboxylic groups for further functionalization (Figure 1a). Before the coating, the PMAO polymer was modified with tetramethylrhodamine 5- and 6-carboxamide (TAMRA) cadaverine, a fluorescent dye to facilitate MNP detection by fluorescence microscopy (MNPs@PMAO-TAMRA).^32,33^ The resulting MNPs were characterized by thermogravimetric analysis (TGA), showing 20% (w/w) oleic acid and 12% (w/w) PMAO (SI, Figure S3). From dynamic light scattering (DLS) and ζ-potential measurements, a hydrodynamic diameter (*D*_H_) of 24.8 ± 4.6 nm and a surface charge of −48 ± 0.4 mV were obtained.

### Functionalization Strategies with a Ready-to-Use NTA-M^2+^ Complex and PEG

As mentioned before, when chelated with divalent metal cations, NTA can react via highly specific noncovalent interactions with histidine-tagged proteins,^22,34^ assuring a proper orientation of recombinant proteins (in this case E/EC12 fragments) on the MNPs. Before performing the bioconjugation, the NTA derivative bis(carboxymethyl)-l-lysine hydrate (LysNTA) was complexed with a divalent metal cation to obtain a ready-to-use LysNTA-M^2+^ complex (Figure 1a, first step). To do this, LysNTA and a salt solution containing the divalent metal ions were mixed for 5 min, and then the mixture was basified with NaOH to remove the excess free metal by precipitation and further centrifugation. This ready-to-use complex is key in our strategy, as it avoids the use of free M^2+^ ions that could lead to MNP precipitation (Figure 3e),^35^ it can be obtained in 20 min, and it is stable for months at 4 °C.

Thereafter, carboxylic groups of water-stable MNPs@PMAO-TAMRA were activated with 1-ethyl-3-(3-dimethyl aminopropyl)carbodiimide) (EDC) in order to bioconjugate two aminated molecules: (i) the ready-to-use LysNTA-M^2+^ complex where Cu^2+^ was used as a proof of concept and (ii) an α-methoxy-ω-amino polyethylene glycol (PEG) with different molecular weights (750 or 5000 Da) for passivating the MNP surface in order to increase their stability in biological media (Figure 1a, step 2).

To incorporate both aminated molecules after activating the carboxylic groups with EDC, two strategies were considered: a one-step strategy and a two-step strategy, hereafter referred to as Strategy 1 or Strategy 2, respectively (Figure 1b). Strategy 1 consisted of carrying out a single reaction in a one-pot fashion, adding at the same time the PEG (18 μmol/mg of Fe) and LysNTA-Cu^2+^ (32 μmol/mg of Fe) molecules. This strategy is fast and easy to carry on, but the number of PEG and LysNTA-Cu^2+^ molecules that are finally incorporated is difficult to control as both molecules are present in the reaction at the same time. On the other hand, Strategy 2 consisted of two consecutive additions of either PEG or LysNTA-Cu^2+^ (the same quantities of aminated molecules were conserved), yielding a more controlled strategy. By alternating the functionalization order between both molecules, two stepwise strategies were set up: strategy 2A where PEG was added in the first place and strategy 2B where LysNTA-Cu^2+^ was added before PEG (Figure 1b).

To corroborate the success of the functionalization process, changes in the electrophoretic mobility of the MNPs after each functionalization step were analyzed by agarose gel electrophoresis (Figure 1c). MNPs@PMAO-TAMRA have a net negative charge due to the carboxylic acid groups present in the PMAO coating and thus migrate toward the positive pole (lane 1). When PEG was added in a first step (strategy 2A, lane 2), the electrophoretic mobility of the MNPs diminished when compared to the control MNPs@PMAO-TAMRA. This variation could be attributed to the increase in the size of the MNPs after they have been functionalized with PEG, as well as to the partial neutralization of the negative charge when carboxyl groups on the MNP surface are replaced with PEG-methoxy molecules. When LysNTA-Cu^2+^ was added to these MNPs in a second step, no further changes were observed in the mobility (strategy 2A, lane 3). This result can be explained by the small size of the LysNTA-Cu^2+^ and its negative charge at the pH under which the gel electrophoresis was performed (pH 8), where a release of the metal ions is promoted by the ethylenediaminetetraacetic acid (EDTA) present in the electrophoresis buffer.^36^ Consequently, both the negative charge on the MNP surface and their size remained similar to those obtained after the first step (introduction of the PEG molecules).

In the other stepwise strategy (2B), in which the first functionalization step was performed by adding LysNTA-Cu^2+^ (lane 6), there was no significant change in the electrophoretic mobility compared to that of control MNPs (lane 1). This is in line with the results discussed above for the other stepwise strategy. However, when in a second step PEG molecules were added to these MNPs (lane 4), a decrease in the mobility was observed, indicating a successful functionalization with this molecule. Interestingly, the mobility never reached that of the MNPs only coated with PEG (lane 2). This suggests that LysNTA-Cu^2+^ was successfully incorporated in the first step, and consequently fewer free carboxylic groups remained available for further reaction with PEG molecules.

Finally, in the case of strategy 1 in which both PEG and LysNTA-Cu^2+^ were added at the same time (lane 5), an electrophoretic pattern similar to the one obtained with MNPs functionalized with strategy 2B (lane 4) was observed. The results are concordant, as when both molecules are added at the same time, the reaction of LysNTA-Cu^2+^ should be kinetically favored due to its smaller size and higher diffusion rate. This results in a PEG grafting similar to that of the stepwise strategy 2B where LysNTA-Cu^2+^ is added in the first step. Although these results suggest that both molecules were incorporated using the one pot strategy, successful functionalization with LysNTA-Cu^2+^ was confirmed in the next step of the bioconjugation procedure, when cadherin was incorporated (*vide infra*).

All these results were also corroborated by evaluating the changes in the surface charge by ζ-potential (Figure 1d) and hydrodynamic diameter by DLS measurements (Tables S1 and S2), respectively.

### Oriented Immobilization of E/EC12 Fragments on the MNPs

**Figure 2:**
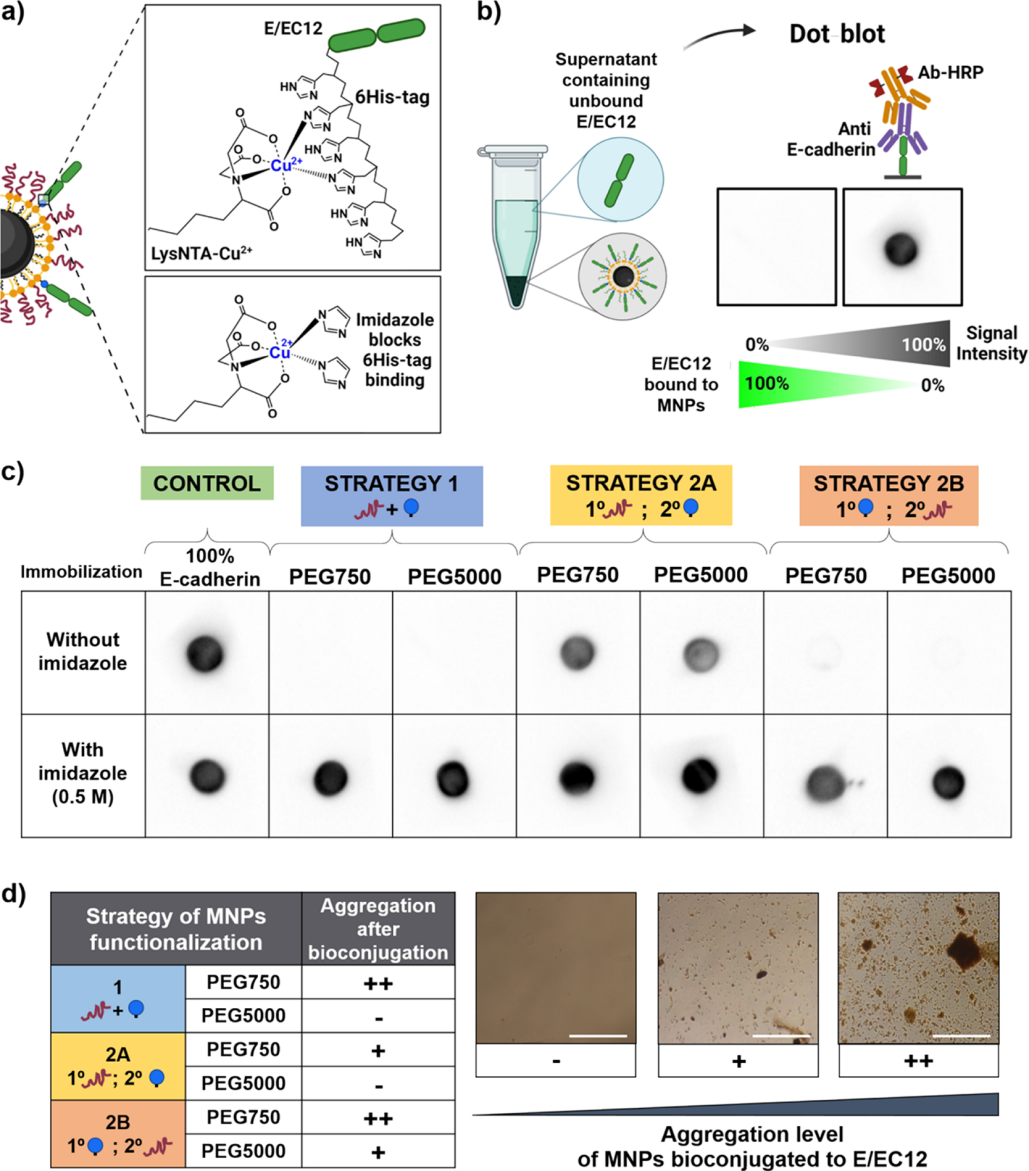
(a) Schematic representation of the E/EC12 fragments binding to LysNTA-Cu^2+^ present on the MNPs through the 6His-tag; imidazole competes with the His-tag, avoiding the bioconjugation. (b) Scheme of the quantification of unbound E/EC12 recovered from supernatants after bioconjugation (by dot blot). (c) Semiquantitative dot blot showing the unbound E/EC12 recovered after the bioconjugation. Strategy 2A where PEG was added in the first place did not bind all protein added, while Strategies 1 and 2B immobilized all protein added, independently of the PEG grafted (750 or 5000 Da). In none of the three strategies the immobilization took place in the presence of 0.5 M imidazole. (d) Aggregation of the MNPs bioconjugated with E/EC12 fragments after 24 h of incubation in PBS. The aggregation level was assessed by optical microscopy and ranked in three levels (no aggregation (−); presence of small aggregates (+); presence of large aggregates (++)); scale bar: 100 μm.

The next step in the functionalization process was the bioconjugation of the E/EC12 fragments to LysNTA-Cu^2+^ present on the surface of the MNPs via metal chelate affinity (Figure 1a, step 3). In this step, the oriented immobilization of the protein fragments on the MNP surface is essential to subsequently generate and maintain the interaction with cellular cadherins.^13^ To confirm that the immobilization was oriented and taking place through LysNTA-Cu^2+^ and the His-tag of the E/EC12 fragments, the bioconjugation was conducted in the presence or the absence of imidazole (0.5 M). Imidazole has a higher affinity for the metal ion present in the LysNTA-Cu^2+^ complexes (K_d_:10^–3^ M) than the 6His-tag of the protein (K_d_:10^–6^ M).^9,37^ Thus, it acts as a competitor, preventing the E/EC12 fragments from binding to the MNPs through their His-tag (Figure 2a).

After the E/EC12 immobilization (using a protein/MNP ratio of 200 μg/mg Fe), MNP@E/EC12 bioconjugates were centrifuged, supernatants containing the unbound protein were collected, and the amount of unbound E/EC12 was analyzed by a semiquantitative dot blot immunoassay (Figure 2b). The samples were compared to a control sample containing the same amount of E/EC12 that was incubated with MNPs (100% of E/EC12). Because the supernatant contains only unbound protein, the signal intensity in the dot-blot analysis is inversely proportional to the amount of protein bioconjugated to the MNPs (Figure 2b). Therefore, this is a specific and reliable way to analyze the amount of protein bound to the MNPs, allowing us to select the best strategy to continue with.

The dot blot results (Figure 2c) indicate that larger amounts of cadherin were conjugated to the MNPs in the cases where LysNTA-Cu^2+^ was added either in the first step of the functionalization (strategy 2B) or in the one-pot strategy (strategy 1), independent of the PEG length. This is consistent with the fact that a large number of LysNTA-Cu^2+^ complexes can be functionalized on the MNPs surface when PEG is not present (strategy 2B), or when PEG is added at the same time (strategy 1), allowing for a higher degree of immobilization of E/EC12 (practically no signal in the supernatant). Conversely, MNPs functionalized by strategy 2A incorporated fewer LysNTA-Cu^2+^ molecules since fewer COOH groups were available after PEG functionalization. Thus, fewer protein molecules could be attached to the MNP surface and a large amount of unbound E/EC12 fragments was present in the supernatant.

In all cases, the attachment of E/EC12 was negligible in the presence of imidazole, demonstrating that the binding was taking place via chelation of the His-tag of the protein with the metal ion and that a competitor such as imidazole blocks the interaction. We can therefore conclude that the E/EC12 fragments were bound in an oriented fashion, which is important for the subsequent binding with cellular cadherins.

In view of these results, we concluded that strategy 2A in which PEG was added in the first place was less efficient for the binding of E/EC12 to the MNP surface and was discarded. We then tested the stability of the MNPs in a physiological buffer (phosphate-buffered saline, PBS), finding out that for the remaining two strategies, passivation with PEG 5000 was always more efficient than with PEG 750 in terms of avoiding the formation of aggregates. No aggregation was found using an optical microscope for MNPs functionalized using strategy 1 or 2B when PEG 5000 was present (Figure 2d). In view of the simplicity and time savings of a one-step reaction, we decided to continue only with strategy 1, using just the longest PEG chains (5000 Da) to ensure a better surface passivation.

As mentioned before, Cu^2+^ ions were selected as a proof of concept to synthesize our LysNTA-M^2+^ complexes. Other metal ions often used for purification of His-tagged proteins are nickel, zinc, and cobalt. The specificity and histidine binding capacity of these divalent cations are different, with the Cu^2+^ cation having the highest protein affinity.^20^ However, Ni^2+^ and Co^2+^ are more commonly used in resins for the purification of His-tagged proteins. To test the versatility of our procedure, bioconjugation was also carried out using Ni^2+^ or Co^2+^ to obtain the LysNTA-M^2+^ complexes. In both cases, the protein immobilization yield remained practically constant and very similar to the one obtained with Cu^2+^ (SI, Figure S4a). Once it was confirmed that the immobilization of cadherins using Ni^2+^ followed the same trend observed with Cu^2+^ for all three strategies (1, 2A, 2B) (SI, Figure S4b), the following assays were only performed using the one-pot strategy and Ni^2+^, due to its widespread use for protein separation purposes.

### Versatility of the Functionalization Strategy: Modulation of the Quantity of LysNTA-Ni^2+^ Functionalized on the MNPs

As mentioned before, the amount of protein present on the surface of the MNPs can be critical to further establish a correct interaction with cellular membrane proteins. Because E/EC12 fragments were immobilized via LysNTA-Ni^2+^ complexes, we hypothesized a direct correlation between the quantity of LysNTA-Ni^2+^ complexes functionalized on the MNPs and the amount of E/EC12 fragments that could be bound. To modulate the amount of LysNTA-Ni^2+^ complexes functionalized on the MNPs using our one-pot strategy, we kept the PEG:MNP ratio constant (18 μmols/mg Fe) and varied the amount of LysNTA-Ni^2+^ complexes added during the functionalization (4, 8, 16, and 32 μmols/mg Fe).

**Figure 3:**
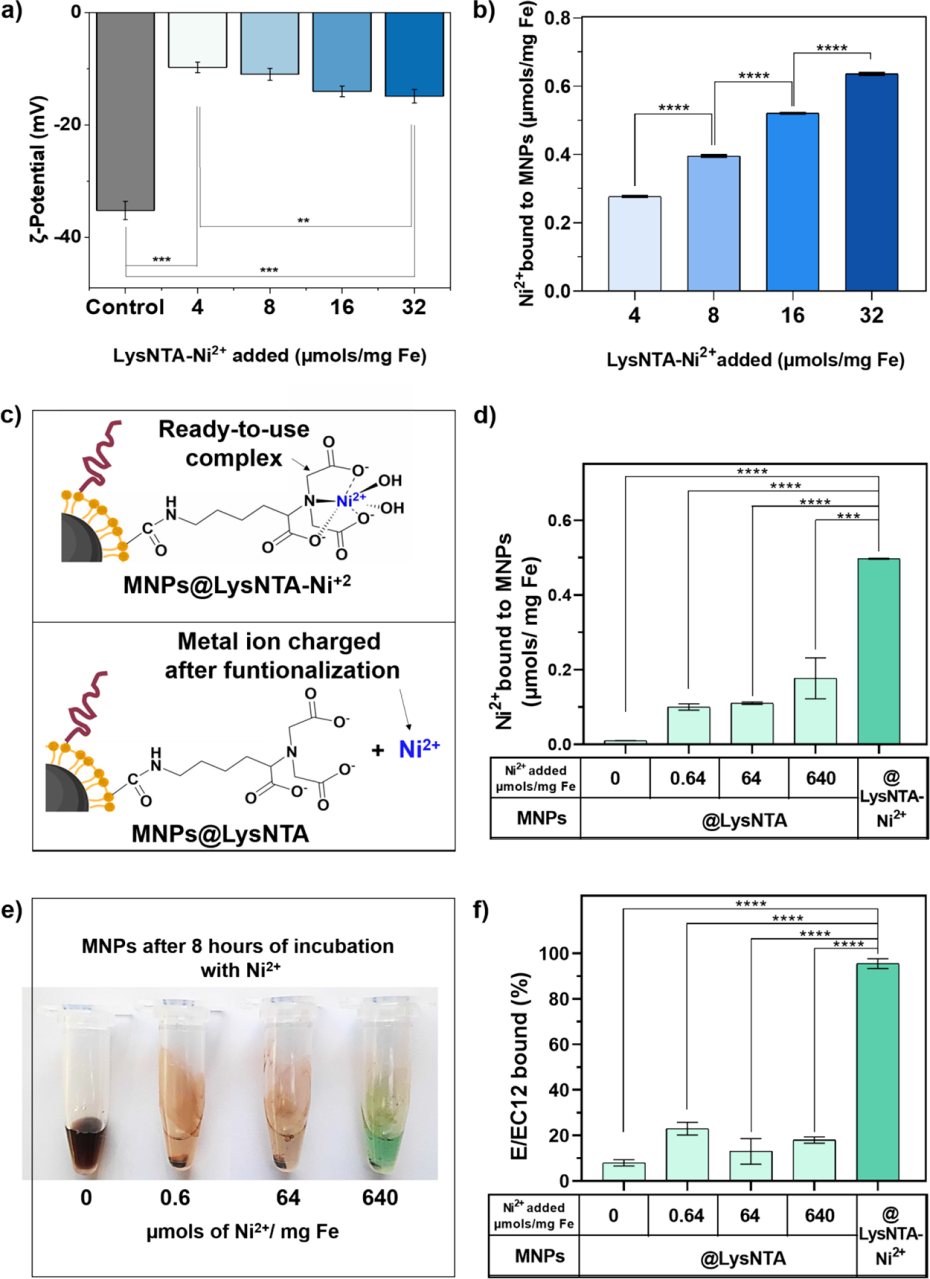
(a) ζ potential measurements of MNPs functionalized with PEG and different quantities of LysNTA-Ni^2+^ (4, 8, 16, and 32 μmols/mg Fe), *N* = 5. (b) ICP-AES analysis performed on MNPs functionalized with PEG and four quantities of LysNTA-Ni^2+^ (4, 8, 16, and 32 μmols/mg Fe); *N* = 2. (c) Scheme of LysNTA-Ni^2+^ functionalization strategies. (d) Quantity of nickel coordinated on MNPs@LysNTA (where different amounts of Ni^2+^ were added afterward) or on MNPs@LysNTA-Ni^2+^ using our ready-to-use complex (32 μmols/mg Fe); *N* = 2. (e) MNP@LysNTA after 8 h of incubation with different quantities of NiCl_2_·6H_2_O; when the highest amount of NiCl_2_·6H_2_O was added, a green color derived from free Ni^2+^ could be observed. This evidences that the amount of Ni^2+^ added was in large excess when compared to the NTA functionalized on the MNPs surface. (f) Percentage of E/EC12 immobilized (200 μg/mg Fe) on MNPs@LysNTA where different amounts of Ni^2+^ were added or on MNPs@LysNTA-Ni^2+^; protein quantification was performed in quadruplicate using a colorimetric Bradford assay. *N* = 2. Black asterisks indicate statistical differences (**p < 0.01; ***p < 0.001; ****p < 0.0001). For panels (a) and (b): one-way ANOVA followed by Tukey’s multiple comparison test; for panels (d) and (f): one-way ANOVA followed by Dunnet’s test.

When compared with control MNPs, all functionalized MNPs showed an increase in the hydrodynamic diameter (SI, Table S3), confirming the PEG grafting in all cases. Furthermore, the ζ-potential measurements shown in Figure 3a revealed a correlation between the MNP charge and the amount of LysNTA-Ni^2+^ added in the functionalization process. The functionalization was also assessed by a TGA analysis. The weight loss variations of MNPs functionalized with LysNTA-Ni^2+^ and PEG molecules were determined mainly by the quantity of PEG molecules present on the MNPs, given their high molecular weight compared with LysNTA-Ni^2+^ molecules. As seen in the Supporting Information (Figure S5), the lower the quantity of LysNTA-Ni^2+^ added (4 μmols/mg Fe), the higher was the weight loss; in other words, the quantity of PEG molecules attached to the MNPs was higher. This result supports the previous hypothesis of a competition mechanism between the PEG chains and LysNTA-Ni^2+^ molecules during one-pot functionalization.

To effectively quantify the LysNTA-Ni^2+^ incorporated on the MNPs, we further analyzed the iron and nickel contents by inductively coupled plasma atomic emission spectroscopy (ICP-AES). As suggested by the other techniques, a direct correlation between the amount of LysNTA-Ni^2+^ added during the functionalization process and the quantity of Ni^2+^ incorporated on the MNPs was found (Figure 3b). In conclusion, we developed a one-step modulable procedure to functionalize MNPs with LysNTA-Ni^2+^ complexes that can be used to bioconjugate His-tag proteins.

### Ready-to-Use LysNTA-Ni^2+^ Complexes Allow a More Efficient Bioconjugation of His-Tag Proteins

Magnetic microparticles are widely used to purify His-tag proteins via metal chelate affinity. This procedure was further extended to MNPs, and several works describe the possibility to modulate the amount of His-tag proteins bound to MNPs.^19,26,38^ However, in the great majority of the cases, the MNPs are first modified with NTA, and the metal ions are added in a second step, often resulting in time-consuming procedures. For instance, Wu *et al.* reported a procedure to obtain MNP-NTA-Ni^2+^ complexes requiring a total reaction time of 12 h, 4 for the functionalization of the MNPs with NTA and 8 for the complexation of Ni^2+^ with the NTA.^26^ Similarly, Xie *et al.* described a protocol implying 6–8 h for the preparation of magnetic Fe_3_O_4_/Au–NTA–Ni^2+^ nanoparticles in a two-step fashion.^38^ Furthermore, these strategies might lead to MNP aggregation when positive metal ions are added to negatively charged MNPs. For instance, Kim et al. reported the formation of aggregates in TEM images after the nickel charging on MNPs containing mono or bis-NTA ligands on their surface.^35^ Lastly, this traditional method can also trigger a cross-linking of the LysNTA molecules during the EDC coupling since LysNTA has both amine and carboxylic acid groups available as potential reactive units.^39^ Precomplexing LysNTA with metal ions prior to the functionalization step might help mitigating undesired cross-linking by blocking its carboxyl groups and avoiding their activation by EDC.^40^

To corroborate if our strategy of preforming the LysNTA-Ni^2+^ before the MNP functionalization could yield better results than the traditional one, we functionalized the MNPs with the same amount of LysNTA (32 μmols/mg Fe), with or without being precomplexed with Ni^2+^ (Figure 3c). For the traditional approach, once the MNPs were modified with LysNTA (MNPs@LysNTA), we incubated them with different amounts of Ni^2+^ (NiCl_2_·6H_2_O). The amounts of Ni^2+^ were selected starting with that corresponding to the highest amount of Ni^2+^ effectively functionalized on the MNPs when using the precharged LysNTA-Ni^2+^ of our strategy (0.64 μmol/mg Fe, as determined from ICP-AES analysis in the previous experiment, Figure 3b). This amount was then increased 100 and 1000 times, respectively, to ensure the availability of metal ions for chelation with LysNTA. The amount of Ni^2+^ incorporated with both strategies was then quantified by ICP-AES, as previously described (Figure 3d).

After 1 h of incubating the MNPs@LysNTA with different amounts of NiCl_2_·6H_2_O, ICP-AES analysis revealed that the amount of metal chelated on the MNPs was significantly lower for all the conditions tested when compared to the MNPs functionalized with our precharged complex. In fact, the highest Ni^2+^ loading that could be reached was three times lower than the one achieved with our ready-to-use LysNTA-Ni^2+^ complex. We further tested whether extending the reaction times to 8 h could lead to a higher Ni^2+^ loading, but during that time of incubation all MNPs lost their stability and aggregated (Figure 3e). We also performed the one-pot functionalization doubling the amount of LysNTA-Ni^2+^ or LysNTA added (64 μmol/mg Fe), while keeping constant the amount of PEG molecules. Although it was possible to incorporate a higher quantity of nickel than when 32 μmol/mg Fe of LysNTA was used, the colloidal stability of the MNPs was compromised, correlating the loss of stability to the higher amounts of nickel added (SI, Figure S6).

Nevertheless, to compare the efficiency of E/EC12 bioconjugation using the two complexation strategies, the protein immobilized on the MNPs functionalized with LysNTA-Ni^2+^ or LysNTA (32 μmol/mg Fe) was also quantified (Figure 3f). As expected, the percentage of bound protein when using the traditional method of adding the metal ions after the MNP functionalization with LysNTA did not reach 30%, while with our method, we were able to bioconjugate more than 90% of the E/EC12 fragments added.

In conclusion, our ready-to-use LysNTA-M^2+^ complex allows a more efficient binding of proteins on the surface of the MNPs when compared to the classical approach based on the complexation of the metal ions with LysNTA immobilized on the MNPs, while also avoiding aggregation. So far, a similar strategy using precharged complexes has only been used for gold nanoparticles.^39,41^ For instance, Abad *et al.* bound horseradish peroxidase (HRP) and ferredoxin-NADP^+^ reductase to gold nanoparticles conserving the enzymatic activity after the conjugation.^41^ In the case of MNPs, the closest example of using a preformed NTA-M^2+^ complex is the work of Lim et al., who linked commercial phospholipids containing a precomplexed NTA directly on the oleic acid shell of MNPs.^21^ However, the resulting MNPs showed aggregation by TEM images, attributed to the sample preparation method but not confirmed using other techniques.

### Tuning the Density of Protein Fragments Functionalized on the MNPs

To corroborate that the amount of Ni^2+^ present on the MNPs has a direct influence on the amount of protein that can be bound, MNPs functionalized with different amounts of LysNTA-Ni^2+^ were bioconjugated with 200 μg of E/EC12 per mg of Fe. The bioconjugation was performed in the presence or absence of imidazole (0.5 M) to examine the possible contribution of nonoriented immobilization.^37^

**Figure 4:**
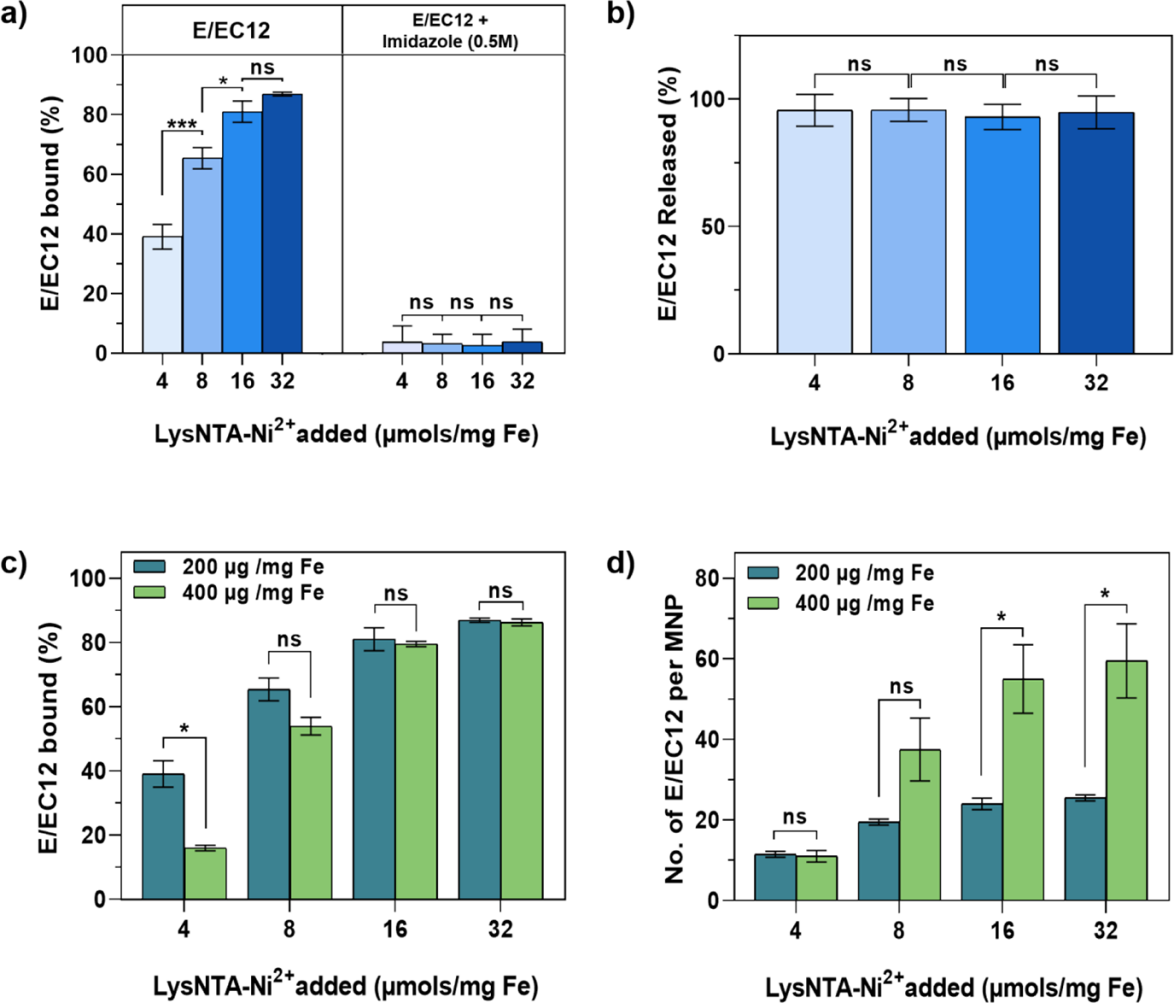
(a) Percentage of E/EC12 immobilized (200 μg/mg Fe) on MNPs functionalized with PEG and four quantities of LysNTA-Ni^2+^ (4, 8, 16, and 32 μmol/mg Fe) in the presence or absence of imidazole (0.5 M) *N* = 2. (b) Percentage of E/EC12 eluted from bioconjugates after incubation with imidazole (0.5 M) *N* = 2. (c) Percentage of E/EC12 immobilized (200 and 400 μg/mg Fe) on MNPs functionalized with PEG and four quantities of LysNTA-Ni^2+^ (4, 8, 16, and 32 μmol/mg Fe) *N* = 2. (d) Calculation of the number of E/EC12 fragments immobilized per MNP (SI, Calculation S1). Black asterisks indicate statistical differences (ns: nonsignificant, *p < 0.05; **p < 0.01; ***p < 0.001). For panels (a) and (b): one-way ANOVA followed by Tukey’s multiple comparison test; for panels (c) and (d): multiple *t* tests.

As shown in Figure 4a, the percentage of E/EC12 immobilized on MNPs functionalized with the lowest quantity of LysNTA-Ni^2+^ complex (4 μmol/mg of Fe) was rather low (38%). This indicates saturation of the LysNTA-Ni^2+^ complexes present on the surface of the MNPs when a high amount of cadherins is offered. On the contrary, MNPs functionalized with higher quantities of the complex (8, 16, or 32 μmol/mg Fe) showed higher percentages of E/EC12 immobilization, demonstrating a clear relationship between the percentage of E/EC12 binding and the quantity of LysNTA-Ni^2+^ present on the MNP surface. When imidazole was added during the functionalization process, E/EC12 was not immobilized in any of the cases. This bioconjugation method thus allows modulation of the amount of proteins/MNP while obtaining a correct orientation of the proteins through chelation of their His-tags with the nickel ions.

To further confirm a specific and reversible bioconjugation through the histidine tag, the MNPs were treated with imidazole following the E/EC12 immobilization.^20^ If upon imidazole addition the E/EC12 fragments remain immobilized on the MNPs, it would demonstrate that they are attached via other unspecific mechanisms and probably without proper orientation.^20,37,42^ The bioconjugates were centrifuged after being incubated with imidazole, and the supernatants containing the released E/EC12 fragments were analyzed. The percentage of E/EC12 displaced from the MNPs was at least 90% for every case (Figure 4b), demonstrating the predominance of E/EC12 oriented immobilization.

To investigate if the amount of cadherin fragments functionalized on the MNPs could be further increased, we performed the bioconjugation using a higher ratio of 400 μg E/EC12 per mg Fe. The data in Figure 4c suggest that MNPs containing the lowest amount of LysNTA-Ni^2+^ (4 μmol/mg Fe) experienced a decrease in the immobilization percentage nearly to half when the amount of protein was doubled. This indicates that the E/EC12 immobilized remained constant due to the saturation of all of the LysNTA-Ni^2+^ moieties present on those MNPs. On the other hand, MNPs functionalized with higher quantities of complexes (8, 16, 32 μmol/mg Fe) did not reach saturation, and higher amounts of protein (>500 μmol/mg Fe) would be needed to reach it (SI, Figure S7).

Calculations were performed to estimate the number of E/EC12 fragments per MNP for each bioconjugate (SI, Calculation S1). As shown in Figure 4d, we were able to tune the number of E/EC12 fragments per MNP from 10 to 60 by varying the amounts of LysNTA-Ni^2+^ and of protein used in the functionalization. Bioconjugates with the lowest and the highest number of E/EC12 fragments/MNP (hereafter referred to as low or high E/EC12 density) were selected for further experiments. These bioconjugates were obtained by adding 400 μg of E/EC12 fragments/mg Fe to MNPs containing the lowest or highest amount of LysNTA-Ni^2+^ (4 or 32 μmol/mg Fe respectively).

### Stabilization of E/EC12 Fragments by Covalent Coupling

**Figure 5:**
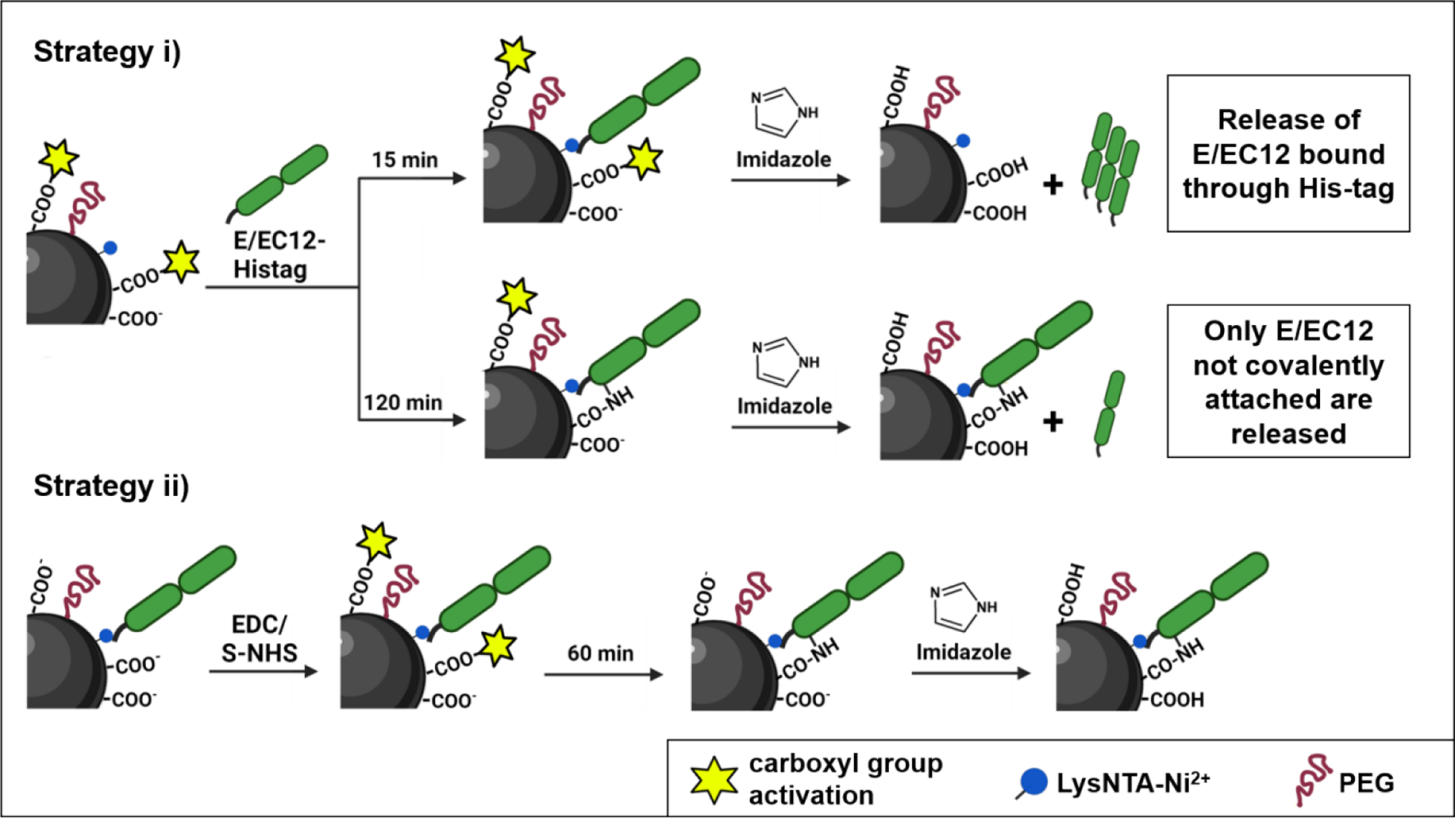
Schematic representation of the two strategies used to covalently immobilize cadherin fragments once the orientation is secured by metal affinity binding. In strategy (i), the carboxylic groups are activated with EDC/S-NHS before adding the proteins, while in strategy (ii), EDC/S-NHS activation of the available COOH moieties is carried out after the E/EC12 fragments are immobilized in an oriented way on the MNPs via metal coordination. In all cases, imidazole is added to elute the protein not bound irreversibly.

Despite the fact that the NTA-[6His-tag] bond mediated by divalent cations such as Ni^2+^ is reasonably stable, it is a noncovalent bond with an affinity in the micromolar range that can become kinetically labile under certain pH or ionic strength conditions.^43−45^ Bearing this in mind, we explored the covalent bonding of the cadherin fragments to the MNPs once their initial orientation had been secured through metal affinity (Figure 5). For this purpose, we took advantage of the carboxyl groups that remain available after functionalizing the MNPs with Lys-NTA-Ni^2+^ and PEG, promoting their site-specific covalent binding to amino groups located in the protein region close to the His-tag.

In a first strategy, these available carboxylic groups were activated using EDC and sulfo-*N*-hydroxysuccinimide (S-NHS) prior to the binding of the His-tagged E/EC12 fragments through metal affinity. To do this, after the activation of the carboxylic groups, the excess of EDC/S-NHS was removed and afterward cadherin fragments were added to the activated MNPs (Figure 5, strategy (i)). Since protein binding through adsorption or affinity phenomena is faster than covalent binding, we hypothesized a two-step binding mechanism, similar to what has been reported to promote site-specific covalent binding of antibodies to MNPs^46,47^ and His-tagged proteins to surfaces.^48−50^ In the first minutes of the reaction, there will be a fast binding of the proteins to the NTA-Ni^2+^ through the His-tag. Subsequently, at longer incubation times, a covalent reaction will occur between the previously activated carboxylic groups and the amino groups present on the already oriented cadherin fragments.

To validate this hypothesis, we evaluated the immobilization of cadherin fragments at two different times, 15 min and 2 h after activation with EDC/S-NHS. As expected, protein immobilization was almost complete at both time points (independently of the presence of EDC/S-NHS) (SI, Figure S8a). To demonstrate that this fast initial protein immobilization takes place through the His-tag via metal coordination and that covalent immobilization occurs at longer times, we added 0.5 M imidazole and quantified the proteins eluted. Proteins bound to the MNPs only through the His-tag should be removed, while those covalently bound should remain immobilized. As can be seen in Figure S8b (Supporting Information), all protein bound in the 15 min sample was released from the MNPs upon addition of imidazole, regardless of whether the MNPs were previously activated with EDC/S-NHS or not. This shows that the first binding occurs through the His-tag (the protein is thus oriented) and that covalent bonds require more time to be established. Incubation for 120 min led to a reduction of the percentage of eluted cadherins, with 60% of the molecules remaining covalently bound. This irreversible attachment only occurred when the samples contained EDC/S-NHS, since in nonactivated MNPs all the protein could be eluted with imidazole.

In a second strategy (Figure 5, strategy (ii)), EDC/S-NHS activation of the available COOH moieties was carried out after the E/EC12 fragments were immobilized in an oriented way on the MNPs via metal coordination. Once again, to confirm the formation of these covalent bonds, we quantified the proteins after imidazole elution. By adjusting the EDC/S-NHS amount added to the reaction, the extent of protein irreversibly bound could be modulated from 5 to 95% (SI, Figure S9).

### Functionality of the MNP@E/EC12 Bioconjugates

**Figure 6:**
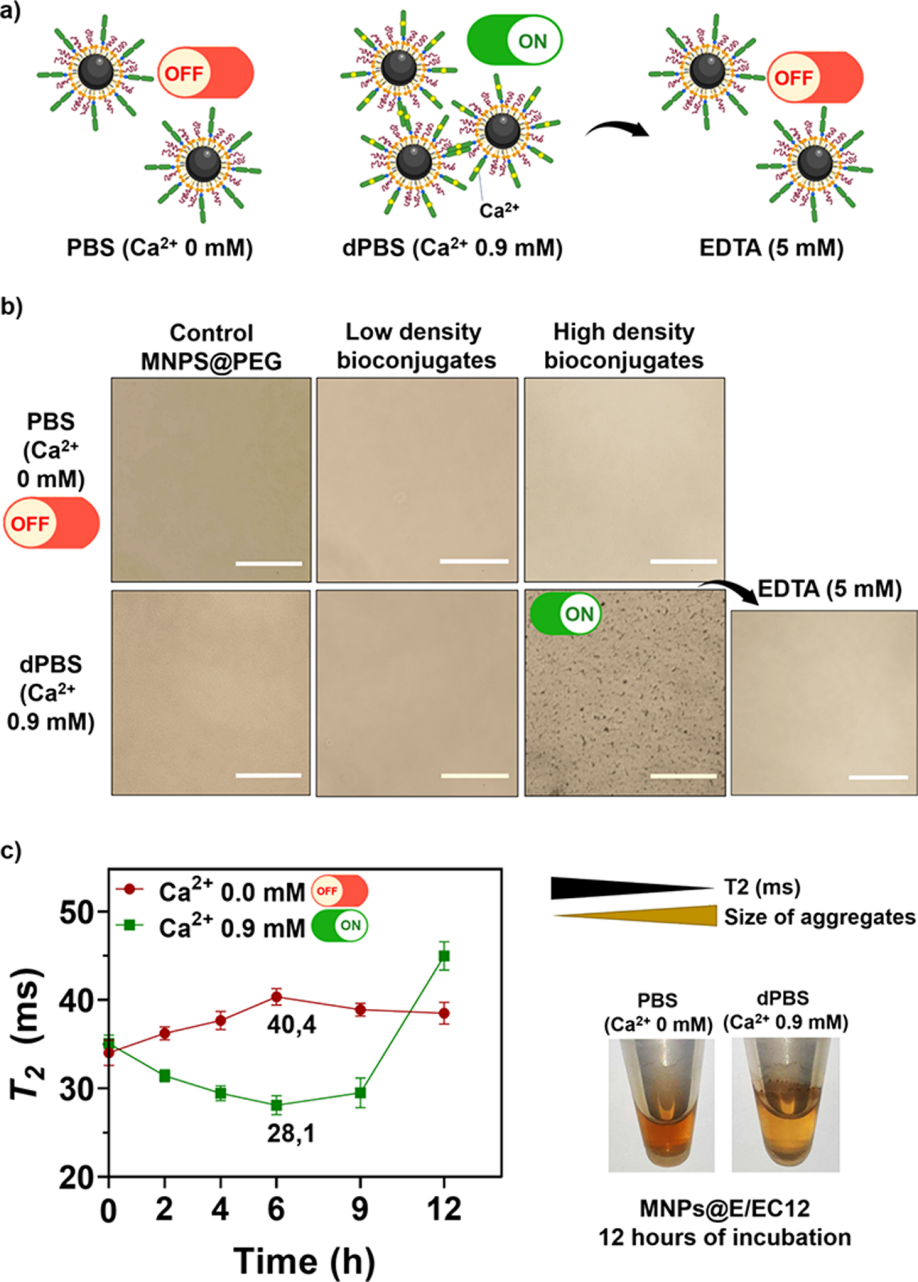
(a) Schematic representation of the MNP bioconjugate aggregation in the presence or absence of calcium ions and upon the addition of EDTA (final concentration: 5 mM). (b) Incubation of bioconjugates with high and low E/EC12 density in buffer with or without calcium, showing the reversibility of aggregation after EDTA (5 mM) addition; scale bars: 50 μm. (c) *T*_2_ (ms) relaxation times of bioconjugates in the presence or absence of calcium ions at different times: the lower the *T*_2_ value, the larger the size of aggregates. After 12 h of incubation, aggregation was observed only in the presence of calcium.

Aside from ensuring correct and stable immobilization of the E/EC12 fragments, it is also critical to verify that these proteins are still functional after their attachment on the MNP surface. To do so, we adapted an aggregation assay previously reported to test cadherin homophilic interactions using microbeads.^51,52^ The assay relies on the calcium-dependent interaction between E-cadherins, which are functional only after a conformational change promoted by the binding of calcium ions (Figure 6a). The formation of MNP aggregates in the presence of calcium indicates successful interaction and functionality of the proteins present on the MNPs.

In nature, the low affinity of intercellular adhesion through cadherin-cadherin binding is addressed by promoting multiple points of adhesion. Therefore, it is expected that the density of cadherin fragments on MNPs is a crucial factor in achieving this type of multipoint binding. Hence, the optimal cadherin density on the MNPs was initially determined with this assay by using MNPs where cadherins were not covalently attached. Bioconjugates with a low or high E/EC12 density were incubated in the presence or absence of calcium ions using PBS (w/o Ca^2+^) or dPBS (Ca^2+^ 0.9 mM) buffer, respectively.^42,53^ As expected, control MNPs functionalized with PEG and LysNTA-Ni^2+^ but not bearing E/EC12, retained colloidal stability in both buffers (Figure 6b). A similar behavior was observed with MNPs functionalized with a low E/EC12 density. Nonetheless, bioconjugates with a high E/EC12 density exhibited aggregation only after incubation in dPBS (Ca^2+^ 0.9 mM), indicating a clear calcium-dependent aggregation.^51^ This result corroborates the importance of the protein density on the MNP surface for the E/EC12 recognition. In addition, the aggregates formed by the bioconjugates with a high E/EC12 density were dissociated after EDTA (5 mM) was added to the solution (Figure 6b). This compound acts as a calcium chelating agent,^54^ preventing the homophilic union between cadherins present on the surface of distinct MNPs. These findings indicate a reversible and calcium-dependent union characteristic of a cadherin-cadherin interaction.^53^

To gain more information about the aggregation dynamics of the bioconjugates, we took advantage of the superparamagnetic nature of the MNPs that can induce local magnetic field inhomogeneities. The presence of MNPs in a solution can change the spin - spin relaxation time (*T*_2_) of the surrounding water protons, and this change can be used for biosensing purposes. Well-dispersed MNPs exhibit different *T*_2_ values compared to aggregated MNPs, making this property useful for detecting changes in the MNP aggregation state.^55^ In our case, the clustering of the MNPs in the presence of Ca^2+^ could be detected by magnetic resonance relaxometry through a decrease in the *T*_2_ relaxation time of water protons.^7,56^ In the absence of Ca^2+^, the *T*_2_ values remained relatively constant (Figure 6c). As expected, when MNPs bearing a high density of E/EC12 fragments were incubated with Ca^2+^ ions, aggregation occurred and a shortening in *T*_2_ values was observed (Figure 6c). *T*_2_ progressively decreased up to 6 h, indicating that aggregate formation took place during this time; after 12 h, aggregation was visible to the naked eye (Figure 6c), which translated into fewer MNPs remaining in solution, and therefore an increase in *T*_2_ values. To study the reversibility of this aggregation, EDTA was afterward added to these samples. The *T*_2_ decreased again to a value closer to that observed at time 0, (SI, Figure S10) corroborating the reversibility of the aggregation observed by the previous aggregation assay.

In the case of bioconjugates in which covalent binding of cadherins was promoted, although they were irreversibly immobilized using the two explored approaches (as described in the previous section), the use of large amounts of EDC/S-NHS in strategy (ii) resulted in a complete loss of protein functionality (SI, Figure S11). This could be attributed to an unintended yet possible covalent cross-linking between the COOH/NH_2_ groups of cadherin fragments bound to different MNPs in the presence of EDC/S-NHS, in contrast to what has been reported with surfaces where protein functionality was preserved using a similar protocol.^44,50^ In contrast, strategy (i) preserves the functionality of cadherins bound to MNPs (SI, Figure S12), thus being a valid strategy for binding His-tagged proteins to MNPs in an orientational and covalent manner if the final application of the bioconjugates so requires.

### Selective Labeling of E-cadherin Expressing Cells

**Figure 7:**
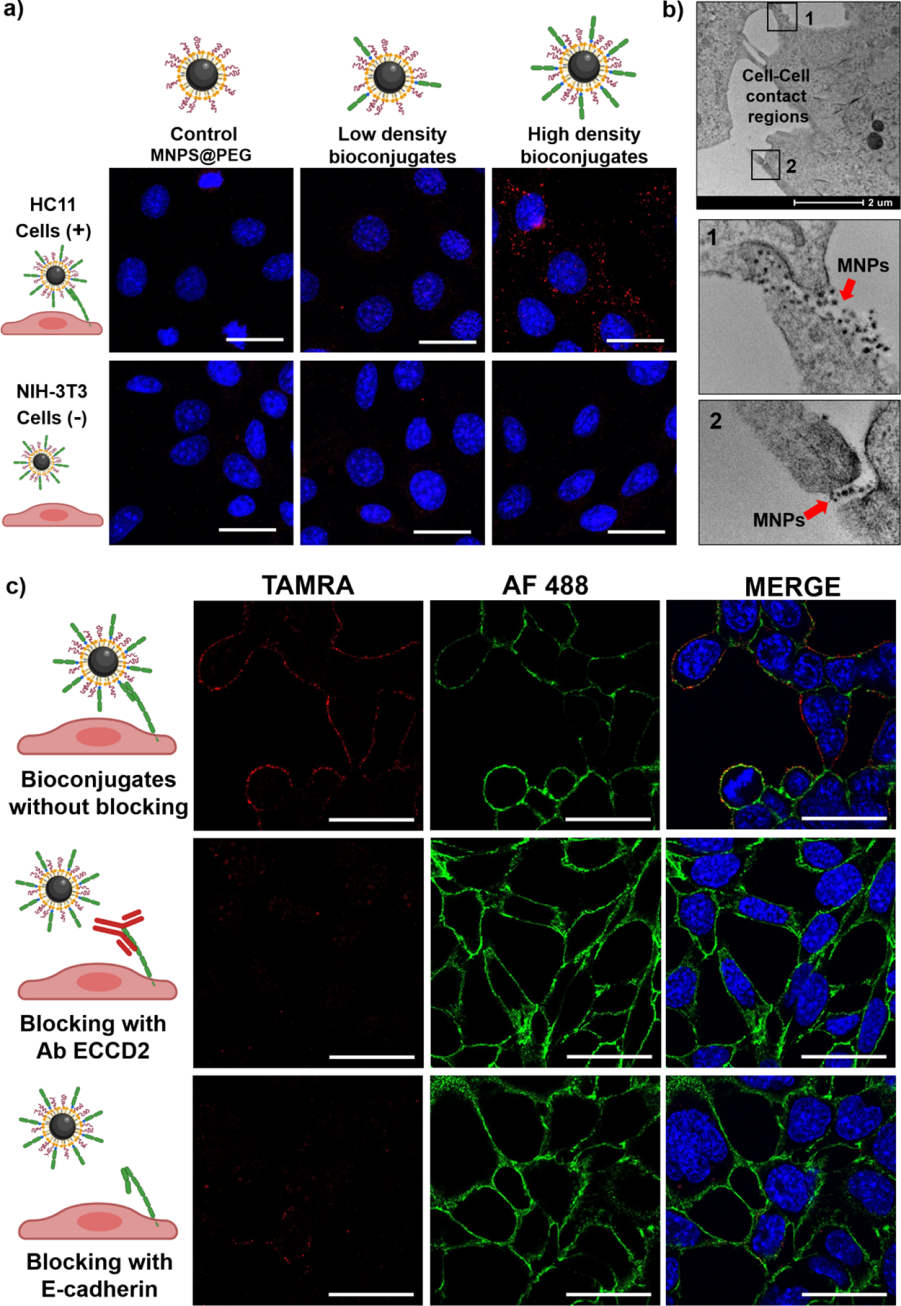
Selective recognition of cells by the MNPs@E/EC12. (a) Confocal microscopy images of mouse epithelial cells (HC11) positive for E-cadherin expression and fibroblasts (NIH-3T3) negative for E-cadherin expression incubated with MNPs@E/EC12 bioconjugates for 3 h at 37 °C; nuclei were stained with 4′,6-diamidino-2-phenylindole dilactate (DAPI; blue); MNPs containing TAMRA are shown in red; scale bars: 50 μm. (b) TEM images of HC11 cells incubated with MNPs@E/EC12 bioconjugates for 3 h. (c) Confocal microscopy images of HC11 cell line incubated with MNPs@E/EC12 bioconjugates; prior to the bioconjugate addition, cells were treated for 1 h at 37 °C with free E/EC12 fragments (50 μg/mL) or ECCD2 antibody (10 μg/mL). Cellular E-cadherins were immunostained and are shown in green (AF488); MNPs containing TAMRA are in red; nuclei are in blue; scale bars: 25 μm. Additional images can be found in the SI (Figure S15).

Selective labeling of cellular E-cadherins was assessed using MNPs decorated with low and high densities of E/EC12 fragments. As a control, MNPs having only a PEG grafting were also used. To test the specificity of the bioconjugates, the E-cadherin expression of mouse epithelial cells (HC11, E-cadherin positive)^57,58^ and mouse fibroblast cells (NIH-3T3, E-cadherin negative)^59^ was first verified by flow cytometry (SI, Figure S13). Cells were then treated with a nontoxic dose (100 μg Fe/mL-SI, Figure S14) of MNPs for 3 h, fixed, and imaged by confocal microscopy (Figure 7a).

As can be inferred from Figure 7a, both HC11 cells (positive for E-cadherin) and NIH-3T3 cells (negative for E-cadherin) did not display a TAMRA signal from control MNPs (PEG) as well as from bioconjugates with low E/EC12 density. Conversely, HC11 cells treated with bioconjugates with a high E/EC12 density exhibited a strong signal arising from the MNPs, while NIH-3T3 cells showed a negligible signal. These findings indicate that only bioconjugates with a high density of E/EC12 fragments on their surface can recognize E-cadherins present on cell membranes, and that the interaction of MNP@E/EC12 bioconjugates is highly specific. To further investigate the cellular localization of the MNPs, the cells were fixed and prepared for observation by TEM. As shown in Figure 7b, MNPs were mainly found in the cell-to-cell contact regions, where E-cadherins tend to accumulate to maintain cell–cell binding.^12,13^

The specificity of the bioconjugates was also evaluated by preincubating the cells with an excess of free E/EC12 fragments that can act as a competitor, or an anti-E-cadherin antibody that recognizes cellular E-cadherin EC1-EC2 domains (ECCD2). As shown in Figure 7c, no MNPs were attached to the cell membrane when the cells were previously treated with the ECCD2 antibody, and a very weak TAMRA signal was observed when cells were previously treated with free E/EC12 fragments. This happens because both molecules interact with the E-cadherins present on the cell, completely blocking or reducing the interaction of the MNPs@E/EC12. However, without blocking, a bright TAMRA signal from the nanoparticles was observed, colocalizing in many cases with cellular E-cadherin stained with Alexa Fluor-488 (AF488) (Figure 7c and SI, Figure S15). All of these results confirmed the specificity of our bioconjugates.

## Conclusions

Protein covalent and oriented immobilization to MNPs is a particularly challenging field as the protein’s functionality needs to be preserved as well as the MNPs stability. In this work, we report a novel and adaptable single-step approach for functionalizing PEG and NTA derivative molecules on MNPs, allowing for fine control of the amount of divalent ions (Cu^2+^, Ni^2+^, Co^2+^) accessible on the MNP surface while conserving the colloidal stability of the MNPs provided by the PEG grafting. By precharging the NTA derivative with the M^2+^ before coupling it to the MNPs, we were able to obtain higher yields of M^2+^ on the MNP when compared to the traditional method where the M^2+^ is added once the NTA is already conjugated to the MNPs. We also show that besides being much faster, this method allows for the controlled bioconjugation of higher amounts of His tag-proteins (E/EC12 fragments modified with His-tag) to the MNPs in comparison to the classical approach. Furthermore, by varying the amount of divalent ions accessible on the MNPs, we can modulate the density of E/EC12 fragments immobilized in an oriented way on the MNPs surface.

The functionality of the bioconjugates was demonstrated by a calcium-dependent and reversible aggregation as well as by specific labeling of cells positive for E-cadherin expression (HC11). E-cadherin is an attractive cellular target due to its implication in many physiological and pathological processes. Its selective targeting with MNPs functionalized with cadherin fragments could allow for the labeling and/or separation of E-cadherin-expressing cells as well as the interrogation of mechanotransduction signals generated by E-cadherin stimulation,^4^ without the need of modifying the cell surface.

The developed method to functionalize MNPs is highly versatile and could be extended virtually to any protein containing a histidine tail as well as adapted to a broad type of different nanomaterials and His-tagged molecules (such as antibodies or enzymes). Aside from orienting the protein on the MNP surface, this method is compatible with maintaining the protein biological function and integrity even if the covalent attachment of the already oriented cadherin is promoted to avoid its release under specific conditions (e.g., acidic pH, elevated ionic strength, or a high concentration of thiolated molecules). Hence, the approach devised here facilitates a broader use of metal affinity interactions for obtaining MNPs that are functionalized with well-oriented and stably bound cadherins. This is particularly valuable for developing applications that operate in complex biological environments.

## Materials and Methods

### Reagents

All commercially available reagents were used as supplied unless otherwise stated. Terrific broth (TB) medium, isopropyl β-D-1-thiogalactopyranoside (IPTG), urea, Na_2_HPO_4_, NaH_2_PO_4_, calcium chloride (CaCl_2_), β-mercaptoethanol, protease inhibitor cocktail, sodium azide, iron(III) acetylacetonate, manganese(II) acetylacetonate, oleic acid (OA), PMAO (MW: 30000–50000 g/mol), 1,2-dihydroxybenzene-3,5-disulfonic acid (Tiron), (Nα,Nα-bis(carboxymethyl)-l-lysine hydrate; LysNTA), CuSO_4_·5H_2_O, NiCl_2_·6H_2_O, Tween 20, bovine serum albumin (BSA), sulfo-*N*-hydroxysuccinimide (S-NHS), anti E-cadherin DECMA-1 antibody (Reference:MABT26), and epidermal growth factor (hEGF) were purchased from Sigma-Aldrich. α-Methoxy-ω-amino poly(ethylene glycol) (PEG, MW: 750 or 5000 Da) were purchased from Rapp Polymere GmbH. Imidazole, benzyl ether, hexane, glycerol, chloroform stabilized with ethanol, 1-ethyl-3-(3-dimethyl aminopropyl)carbodiimide (EDC), Bradford reagent assay, bovine serum albumin standard (BSA) were obtained from Thermo Scientific. Absolute ethanol, sodium hydroxide (NaOH), hydrochloric acid (HCl), nitric acid (HNO_3_), ethylenediaminetetraacetic acid (EDTA), and tris(hydroxymethyl)aminomethane (Tris) were obtained from Panreac. Agarose beads Ni^2+^ superflow (QIAGEN, ref. 30430) were purchased from Quiagen. PD-10 desalting columns packed with Sephadex G-25 resin were obtained from Cytiva. Tetramethylrhodamine 5- and 6-carboxamide (TAMRA) were obtained from AnaSpec. Anti-E-cadherin antibody produced in rabbits for Dot-blot (Reference: SAB5700789) and an Immobilon Western Chemiluminescent HRP Substrate were purchased from Merck. Goat anti-rabbit immunoglobulin/HRP (secondary antibody) was purchased from Dako (Reference: P044801–2). Amicon centrifugal filter units (100 kDa MWCO) and 0.22 μm pore size 13 mm diameter cellulose acetate membrane filters were obtained from Millipore. 4–15% Mini-PROTEAN TGX precast protein gels were purchased from Bio-Rad. Roswell Park Memorial Institute 1640 (RPMI), Dulbecco’s Modified Eagle Medium (DMEM), fetal bovine serum (FBS), 1X glutaMAX, and antibiotic penicillin-streptomycin (10000 U/mL) were obtained from Gibco. Paraformaldehyde and glutaraldehyde were purchased from Electron Microscopy Sciences. E-cadherin monoclonal antibody (ECCD2; reference:^13^-1900), goat Anti-Rat Alexa Fluor 488 (Reference: A-11006), 4′,6-diamidino-2-phenylindole dilactate (DAPI), and prolong Diamond were obtained from Invitrogen. Buffers were prepared according to standard laboratory procedures. Milli-Q water (resistivity of 18.2 MΩ/cm at 25 °C) was obtained using a Milli-Q Advantage A10 system. Mouse epithelial cells (HC11) and mouse fibroblast cells (NIH-3T3) were acquired from ATCC.

### E/EC12 Fragment Expression and Purification

E/EC12 fragments were expressed in the *E. coli* BL21 strain containing a pET plasmid in which a sequence encoding the E/EC12 fragment with a hexahistidine tag at the C-terminal end was cloned. The plasmid was kindly provided by Dr. Helene Feracci (CNRS, Bordeaux, France).^60^ Expression was performed in terrific broth (TB) medium, inducing expression with isopropyl β-IPTG for 2 h at 37 °C. The culture was centrifuged at 5000*g* and 4 °C for 20 min, and the pellets were incubated with urea buffer (4 M urea, 50 mM Na_2_HPO_4_, 20 mM imidazole, 20 mM β-mercaptoethanol and a protease inhibitor cocktail) for 20 min at 4 °C. Thereafter, a centrifugation step was performed at 5000*g* and 4 °C for 20 min to separate the supernatant containing the protein from the cell debris. The protein was incubated with agarose beads containing Ni^2+^ ions on their surface for two h to attach the proteins by their His-tags. The beads were washed six times with urea buffer and were dialyzed against a decreasing urea gradient: 3 M, 2 M, 1 M, performing six dialysis steps with PBS containing β-mercaptoethanol.

The resultant beads containing the E/EC12 fragments were stored at 4 °C in PBS buffer with 0.05% sodium azide. When needed, the proteins were recovered by adding a solution of 0.5 M imidazole to the beads. The remaining imidazole was eliminated using PD-10 desalting columns, leaving the purified protein in PBS (pH 7.4).

Protein fragments were quantified by absorbance at 280 nm, and the concentration was calculated by the Beer–Lamber Law. The online ExPASy-ProtParam tool (https://web.expasy.org/protparam/) was used to predict the molecular weight (MW: 25,205 kDa) and molar extinction coefficient (ε: 0.846) from the amino acid sequence. Samples of 2 μL were used for quantification on a Biotek Synergy H1 UV/vis microplate spectrophotometer.

### Circular Dichroism

Measurements were carried out in a JASCO Circular dichroism Spectropolarimeter model J-810 in the far UV region between 200 and 280 nm with a bandwidth of 1.0 nm, scanning speed of 100 nm/min, and response time of 1 s. A single cuvette with a path length of 0.1 cm was used for all the measurements. Before measurements, the E/EC12 concentration was determined by absorbance at 280 nm. Two E/EC12 solutions of 300 μL in PBS (∼200 μg/mL) were prepared and measured in the absence of calcium ions, and thereafter PBS or a concentrated solution of CaCl_2_ (pH 7.4; 270 mM) was added, leaving a final concentration of calcium of 0.9 mM. The experiment was repeated twice. All spectra were blank corrected.

### Synthesis of Manganese Iron Oxide Nanoparticles

Nanoparticles were synthesized by following a one-step thermal decomposition method. First, 13 mmol of Fe(acac)_3_, 2 mmol of Mn(acac)_2_, and 40 mmol of oleic acid were added to 150 mL of benzyl ether and mechanically stirred under a flow of nitrogen. The mixture was heated to 200 °C for 2 h with a rate of 3 °C/min. After, the reaction temperature was increased to 285 °C with a rate of 5 °C/min and kept at this temperature for 2 h. Finally, the flask was cooled to room temperature under an inert atmosphere. Under ambient conditions, an ethanol/hexane 3:1 excess was added to the mixture, and a black material was isolated via magnetic separation overnight. Afterward, the product was suspended in hexane, precipitated with ethanol, and magnetically separated. This suspension–precipitation cycle was repeated 4 times, resulting in a black hexane/oleic acid dispersion, which was stored at 4 °C for future uses.

### Coating of MNPs with Poly(maleic anhydride-*alt*-1-octadecene) (PMAO)^33^

225 mg of PMAO (MW: 30000–50000 g/mol) were dissolved in 15 mL of CHCl_3_. At the same time, 2 mg of TAMRA was dissolved in absolute ethanol (1 mg/mL) and transferred to the flask containing the PMAO solution, leaving the mixture protected from light and under magnetic stirring at room temperature overnight. 80 mL of CHCl_3_ was added to the mixture, and then 10 mg of Fe of the hydrophobic nanoparticles (previously washed thrice with ethanol and resuspended in 2 mL of CHCl_3_) was added dropwise in an ultrasonic bath. The mixture was kept in the bath for 15 min at room temperature. The excess of CHCl_3_ was evaporated using a rotary evaporator at a pressure of 200 mbar at 40 °C until a final volume of 5–10 mL. Once eliminated the organic solvent, 20 mL of 0.05 N NaOH was added to the mixture to hydrolyze the anhydride groups present in the PMAO and to confer stability in water to the MNPs. The remaining organic solvent was evaporated as before, this time under a pressure of 200 mbar and at 70 °C until a final volume of 10–20 mL. The resulting suspension was filtered using a Millipore filter (0.22 μm) to remove MNP aggregates. The excess of PMAO was eliminated by four ultracentrifugation steps at 70000*g* per 2 h each one, and the resultant nanoparticle suspension MNP@PMAO-TAMRA was stored at 4 °C, protected from light.

### Determination of Iron Concentration

After each coating, functionalization, or immobilization step, determination of iron concentration was performed.^61^ First, 5 μL of MNPs were diluted in 45 μL of solvent (hexane or water) and digested with 100 μL of aqua regia solution (HCl:HNO_3_; 3:1) at 60 °C for 15 min. Then, the samples were diluted up to 500 and 50 μL were used for the iron quantification by mixing the digested samples with 0.25 M 1,2-dihydroxybenzene-3,5-disulfonic acid (Tiron), a molecule that forms a colored complex with iron and can be measured by spectrophotometry (480 nm).^62^ The samples were measured on a Biotek Synergy H1 UV/vis microplate spectrophotometer and compared with a standard calibration curve obtained with solutions of known iron concentrations (0, 100, 200, 400, 600, 800 μg Fe/mL). All of the iron determinations were made in triplicate.

### Gel Electrophoresis

Agarose gels with a concentration of 2% (m/v) were prepared using a tris(hydroxymethyl)-aminomethane, borate, and EDTA (TBE) 0.5× buffer. Samples were mixed with 25% glycerol prepared in TBE (0.5x), and a final volume of 8 μL was charged for the electrophoresis. The gel was run during 60 min at 120 V.

### Preparation of the LysNTA-M^2+^ Complex

An NTA derivative (Nα, Nα-bis(carboxymethyl)-l-lysine hydrate), which contains an extra tail ending in a primary amine group (NH_2_) was used (LysNTA). The complex LysNTA-M^2+^ was obtained by mixing 50 mL of a 25 mM solution of LysNTA with 30 mM of metal salt (CuSO_4_·5H_2_O; NiCl_2_·6H_2_O or CoCl_2_·6H_2_O) in a borate-buffered saline (BBS, 50 mM, pH 8.0) during 5 min. The pH was increased up to 10.5 for Ni^2+^ or Cu^2+^ and 10 for Co^2+^ ions. Finally, the excess of free metals were precipitated with NaOH and discarded by centrifugation at 5000*g*, room temperature for 10 min, the pH was adjusted to 9.0, and the complex was stored at 4 °C.

### Functionalization of MNP@PMAO-TAMRA with PEG and/or LysNTA-M^2+^

MNPs@PMAO-TAMRA (0.5 mg of Fe) was mixed with LysNTA-M^2+^ (4, 8, 16, 20, 32, 64 μmol/mg Fe) and 18 μmol of methoxy-polyethylene glycol-amine (PEG) molecules (MW: 750 or 5000 Da respectively) in a final reaction volume of 1.5 mL. Functionalization was carried out with 1-ethyl-3-(3-dimethyl aminopropyl)carbodiimide (EDC) in BBS (50 mM, pH 9). The mixture was warmed to 37 °C and 20 μmol of EDC was added to the same reaction tube twice, at time 0 and after 30 min, maintaining the mixture under stirring in a horizontal shaker at 800 rpm for three h and 30 min. Then, MNPs were washed with distilled water and centrifugal filters (Amicon, Millipore, 100 kDa cutoff) to eliminate unreacted reagents, and stored at 4 °C. In the case of the two-step functionalizations, a similar procedure was followed but adding only 20 μmol/mg Fe of LysNTA-M^2+^ or 18 μmol/mg Fe of PEG molecules. After washing to eliminate unreacted reagents, the protocol was repeated for the remaining molecule.

### Immobilization of E/EC12 Fragments on MNPs

MNPs (0.1 mg Fe) previously functionalized with LysNTA-M^2+^ and PEG were mixed with E/EC12 fragments (100, 200, or 400 μg/mg Fe) suspended in a phosphate-buffered saline solution (PBS, w/o Ca^2+^ or Mg^2+^) in a final reaction volume of 200 μL. Imidazole (0.5 M) was added to selected samples to evaluate the oriented protein immobilization. The bioconjugation was performed by stirring the microtubes on a horizontal shaker for one h at 37 °C. An extra tube only containing E/EC12 fragments was prepared as the 100% control to quantify the bioconjugation.

To elute the protein fragments from the MNPs surface, the bioconjugates (0.1 mg Fe) were incubated with imidazole (final concentration = 0.5 M) in 200 μL. The incubation was performed by shaking the samples in an orbital stirrer during 1 h at 37 °C. Afterward, the MNPs were centrifuged during 1 h at 20000*g* and 4 °C to separate the released protein for quantification.

### Dot Blot

A drop of 3 μL per sample was added to 1 cm^2^ nitrocellulose membranes and was left to dry for 30 min. E/EC12 incubated without the MNPs (100%) in PBS buffer containing or not imidazole (0.5 M) were used as protein controls. The membrane was blocked with TBST buffer (Tris Buffered Saline (TBS) 1X + 0.1% Tween 20) + 2.5% bovine serum albumin (BSA), during 30 min at 37 °C. After blocking, four washes were performed adding TBST for 5 min at 37 °C. Then, membranes were incubated with anti-E-cadherin CD324 polyclonal antibody in TBST (0.75 μg/mL) for 30 min at 37 °C and four washes were performed again. The procedure was repeated by adding a secondary anti-rabbit antibody-HRP (5 μg/mL), including the washing step. Samples were revealed with a mixture of Immobilon Western Chemiluminescent HRP Substrate of Peroxide solution:Luminol reagent 1:1, and the results were visualized in a Chemidoc (Bio-Rad).

### Bradford Assay

After immobilization of E/EC12 fragments on MNPs, the bioconjugates were centrifuged for 1 h at 17000*g* and 4 °C to separate unbound protein. Supernatants were collected for quantification, while MNPs were resuspended in PBS and stored at 4 °C. Supernatants were quantified by using a Bradford reagent assay following manufacturer’s instructions. Briefly, a working ratio of 1:10 sample:reagent (20:200 μL) was used, and the samples were incubated at room temperature for 10 min before quantification at 595 nm (Biotek Synergy H1 UV/vis microplate spectrophotometer). Each supernatant was quantified in quadruplicate and compared with a standard curve made with known concentrations of bovine serum albumin (BSA).

### Covalent Coupling of E/EC12 Fragments on the MNPs

The covalent coupling was performed using two strategies:

1 MNPs (0.25 mg of Fe) previously functionalized with LysNTA-Ni^2+^ (32 μmol/mg of Fe) and PEG were mixed with 17.5 μmol of EDC, and 26.3 μmols of S-NHS in MES buffer (50 mM; pH 6.5) with a final reaction volume of 900 μL. The mixture was stirred on a rotator for 30 min. The excess of EDC/S-NHS was eliminated using a PD-25 desalting column, leaving the purified MNPs in PBS buffer (pH 7.4). Then, the MNPs were concentrated with centrifugal filters (Amicon, Millipore, 100 kDa cutoff) during 4 min at 6000 g, suspended in PBS and bioconjugated with E/EC12 fragments (400 μg/mg Fe). From the resultant bioconjugates, 0.1 mg of Fe was used to quantify the percentage of protein bound and 0.1 mg of Fe was incubated with imidazole as described before for eluting the E/EC12 fragments noncovalently coupled from the MNP surface. The remaining 0.05 mg of Fe was used to assess the functionality of the bioconjugates.
2 MNPs (0.1 mg of Fe) previously bioconjugated with E/EC12 fragments (400 μg/mg Fe) were mixed with four different ratios of EDC/S-NHS μmol (0.14/0.21; 0.28/0.42; 0.57/0.84; 2.85/4.2) in MES buffer (20 mM; pH 7.0) with a final reaction volume of 1000 μL. The mixture was stirred on a horizontal shaker for 1 h at 37 °C. The bioconjugates were washed with PBS and centrifugal filters (Amicon, Millipore, 100 kDa cutoff) during 4 min at 6000*g* to eliminate the excess of EDC and S-NHS. Then, bioconjugates were suspended in PBS and incubated with imidazole as described in the previous strategy.

Released protein was quantified by the Bradford assay. The functionality of all of the bioconjugates was assessed by the calcium aggregation assay described below.

### Calcium Aggregation Assay

Bioconjugates with a low or high E/EC12 density were used for this experiment. To obtain the bioconjugates, the MNPs functionalized with PEG and LysNTA-Ni^2+^ (4 or 32 μmol/mg Fe) were used as starting material to immobilize onto them 400 μg of E/EC12 fragments/mg Fe. The bioconjugates were filtered using a Millipore filter (0.22 μm diameter) and the resultant MNPs were diluted to 200 μg Fe/mL in PBS (w/o Ca^2+^ or Mg^2+^) or dPBS (0.9 mM Ca^2+^ and Mg^2+^) buffer. 100 μL of each bioconjugate was added to a 96-multiwell plate and were incubated for 12 h in a horizontal shaker at 600 rpm and 37 °C. After this time, the samples were observed under a bright-field microscope Nikon ECLIPSE Ti. The assay was repeated twice. Images were acquired by using the NIS-Elements software.

### Aggregation Evaluated by MiniSpec Measurements

Bioconjugates with a high E/EC12 density were diluted to 100 μg Fe/mL in PBS (w/o Ca^2+^ or Mg^2+^) or dPBS (0.9 mM Ca^2+^ and Mg^2+^) buffers in a final volume of 250 μL. Samples were prepared in duplicate and incubated in a horizontal shaker at 600 rpm and 37 °C. The transversal (*T*_2_) relaxation times were measured in a Bruker Minispec relaxometer at 0.47*T* at different times (0, 2, 4, 6, 9, 12 h). Samples were diluted in the same buffer (PBS or dPBS) to 5 μg of Fe/mL before each measurement in a final volume of 350 μL.

### Cadherin Cellular Targeting

Mouse epithelial cells (HC11) and fibroblast (NIH-3T3) cells were grown in Roswell Park Memorial Institute 1640 (RPMI) and Dulbecco’s Modified Eagle Medium (DMEM) media, respectively, both supplemented with 10% fetal bovine serum (FBS), 1× glutaMAX (Gibco) and antibiotic penicillin-streptomycin (10,000 U/mL). HC11 medium was supplemented with epidermal growth factor (EGF) with a final concentration of 10 ng/mL. HC11 or NIH-3T3 cells were seeded at a density of 13 × 10^4^ or 15 × 10^4^ cells per well respectively and were grown in a 24-multiwell plate for 24 h in a 5% CO_2_ atmosphere and 37 °C. MNPs@PEG (control) and MNPs@E/EC12 bioconjugates were sterilized by filtration with a Millipore filter (0.22 μm diameter), and the resulting MNPs were diluted in RPMI or DMEM medium reaching a final concentration of 100 or 200 μg Fe/mL.

Cells were incubated in RPMI or DMEM medium without FBS for one h at 37 °C. Then, cells were washed once with PBS and incubated in fresh PBS during 5 min at 37 °C. After this time, 200 μL of the MNPs solutions was added to each well and incubated for 1 or 3 h. Afterward, the cells were washed once with dPBS buffer and 200 μL of 2% paraformaldehyde was added to each well and incubated during 10 min at 37 °C to fix the cells. After this time, the cells were washed twice with dPBS buffer.

After fixation, immunofluorescence of cellular E-cadherin was performed. HC11 cells were blocked with dPBS supplemented with BSA 1% during 1 h at RT, washed thrice with dPBS for 5 min, and incubated with a solution of 5 μg/mL of anti E-cadherin DECMA antibody (produced in rat) for 2 h at RT. Cells were washed again thrice with dPBS for 5 min, and the samples were incubated with a solution of 2 μg/mL of goat anti-rat Alexa Fluor 488 for 45 min at room temperature. The cells were washed three times with dPBS for 5 min, nuclei were stained with a DAPI dilactate solution (1 μg/mL) for 8 min at RT, and two final washes were performed with dPBS. Coverslips were mounted using Prolong Diamond. Fluorescence images were acquired using a Zeiss LSM880 confocal laser scanning microscope (Centro de Investigación Biomédica de Aragón (CIBA), Spain) using the software ZEN 3.4 black edition. Image acquisition was performed using three laser wavelengths: 405, 488, and 561 nm.

### TEM Analysis of MNP–Cell Interaction

HC11 cells were seeded (9 × 10^3^ cells per well) onto eight-well chamber slides from Lab-Tek in 300 μL of RPMI and grown for 24 h. Cells were then treated with MNPs@E/EC12 bioconjugates (high density) at 100 μg/mL for 1 h and washed with PBS for 2 min. Cells were fixed with 3% glutaraldehyde in dPBS for 10 min at 37 °C. The fixative agent was replaced with fresh glutaraldehyde and incubated for 2 additional hours at room temperature. Fixed cells were rinsed with phosphate buffer (0.1 M) and kept at 4 °C. Sample sectioning and grid mounting were performed by the Electron Microscopy Service at the Centro de Investigación Principe Felipe (CIPF, Valencia, Spain). Briefly, the samples were postfixed in 2% OsO_4_ for 1 h at room temperature and stained in 2% uranyl acetate in the dark for 2 h at 4 °C. Then, they were rinsed in distilled water, dehydrated in ethanol, and infiltrated overnight in Durcupan resin (Sigma-Aldrich, St. Louis, USA). Following polymerization, embedded cultures were detached from the wells and glued to the Durcupan blocks. Finally, ultrathin sections (0.08 μm) were cut with an Ultracut UC-6 (Leica microsystems, Wetzlar, Germany) and stained with lead citrate (Reynolds solution).

For the visualization, an FEI Tecnai T20 microscope was used at an accelerating voltage of 200 kV (Laboratorio de Microscopías Avanzadas, University of Zaragoza, Spain).

### ICP–AES Analysis

The total iron and nickel concentration were determined by ICP–AES. For ICP measurements, 20 μL of MNP suspensions was treated with 300 μL of HCl (37%) solution for 1 h at 80 °C, and then the digested samples were diluted with Milli-Q water up to 10 mL, and analyzed with a HORIBA Jobin Yvon-ACTIVA-M CCD ICP spectrometer at the SGIker (UPV/EHU) service. Measurements were performed in axial mode with limits of quantification (LOQs) of 0.005 ppm for the two elements. Two wavelengths were used, one to quantify from 0 to 10 ppm and the second to verify the difference between concentrations using both wavelengths was acceptable with an error of <5%. The wavelengths were: for iron 238.204 nm (quantitative) and 259.940 nm (confirmation); for nickel 231.604 nm (quantitative) and 216.555 nm (confirmation). Experiments were carried out in triplicate, and results are represented as the mean value ± the standard deviation.

### Thermogravimetric Analysis

MNPs in organic solvents were air-dried, while MNPs in water were freeze-dried. Thermogravimetric measurements were performed in a Universal V4.5A TA Instrument under an air atmosphere at a flow rate of 50 mL/min at a rate of 10 °C/min until a final temperature of 800 °C.

### Dynamic Light Scattering and ζ-Potential Measurements

Measurements were performed on a Malvern Zetasizer Nano instrument considering a refractive index of 2.0 and an absorption index of 1.0 for Fe_3_O_4_. Samples were prepared at a concentration of 0.05 mg Fe/mL and sonicated 10 s before measurement, the samples were irradiated with a monochromatic helium–neon laser of 633 nm. In the case of the ζ-potential, the dispersed light at an angle of 13° was measured. Each sample was measured five times at 25 °C, combining 10 runs per measurement. Results were treated using the Malvern software Zetasizer Nano 7.13.

### Statistical Analysis

Data were analyzed using GraphPad Prism 6.0 (GraphPad Software, San Diego, USA). The results are represented as the average ± standard deviation of at least two independent experiments. Analysis of variance (ANOVA) one-way with Tukey’s or Dunnet’s comparison test were used to evaluate differences between groups, which were considered statistically significant at a *p* value of <0.05. Simple and multiple *t* tests were also used, which were considered statistically significant at a *p* value of <0.05.

## Supporting Information Available

Characterization of protein fragments (SDS-PAGE and Circular Dichroism), MNPs (TEM, TGA) and bioconjugates (DLS, Z-potential, TGA); comparison of the bioconjugation efficiency when using different metallic ions; functionalization with 64 μmol/mg Fe of LysNTA-Ni^2+^ or LysNTA; saturation of the MNP surface with protein fragments and calculation of the number of fragments immobilized; covalent coupling; analysis of E-cadherin expression in different cell lines, MTT cytotoxicity and cellular labeling (PDF)
