# Supplementary material for: A Simple and Versatile Strategy for Oriented Immobilization of His-Tagged Proteins on Magnetic Nanoparticles
Source: Bioconjug Chem. 2023 Oct 26;34(12):2275–92. doi: 10.1021/acs.bioconjchem.3c00417 (PMC10739578; doi:10.1021/acs.bioconjchem.3c00417)
Supplement: Supplementary file 1 — bc3c00417_si_001.pdf [file bc3c00417_si_001.pdf]

Supporting Information

A simple and versatile strategy for oriented immobilization of His-tagged proteins on magnetic nanoparticles

Christian Castro-Hinojosa,[a] Susel Del Sol-Fernández,[a] Eduardo Moreno-Antolín,[a] Beatriz Martín-Gracia,[a] Jesús G. Ovejero,[b,c] Jesús Martínez de la Fuente,[a,d] Valeria Grazú,[a,d] Raluca M. Fratila [a,d,e] María Moros* [a,d]*

[a] *Instituto de Nanociencia y Materiales de Aragón, INMA (CSIC-Universidad de Zaragoza), C/ Pedro Cerbuna 12, 50009, Zaragoza, Spain.*

[b] *Instituto de Ciencia de Materiales de Madrid (ICMM/CSIC), Sor Juana Inés de la Cruz 3, 28049, Madrid, Spain.*

[c] *Department of Dosimetry and Radioprotection, General University Hospital Gregorio Marañón, Dr Esquerdo 46, 28007, Madrid, Spain*

[d] *Centro de Investigación Biomédica en Red de Bioingeniería, Biomateriales y Nanomedicina (CIBER-BBN), 28029, Madrid, Spain.*

[e] *Departamento de Química Orgánica, Facultad de Ciencias, Universidad de Zaragoza, C/ Pedro Cerbuna 12, 50009, Zaragoza, Spain.*

Corresponding authors: Raluca M. Fratila (rfratila@unizar.es); María Moros (m.moros@csic.es)

Index:

1. E/EC12 cadherin fragments characterization 3

2. MNP characterization 4

3. Characterization of MNPs after functionalization with PEG + LysNTA-M^{2+} 6

4. Comparison of the bioconjugation efficiency by using LysNTA complexed with different metallic ions 8

5. Characterization of MNPs after functionalization with PEG and different quantities of LysNTA-Ni^{2+} 9

6- Functionalization with LysNTA or ready-to-use LysNTA-Ni^{2+} complexes 11

7. Saturation of the MNP surface 12

8. Calculation of the number of E/EC12 fragments immobilized/MNP 13

9. Covalent coupling 15

10. Assessment of the MNP@E/EC12 bioconjugate functionality by magnetic resonance relaxometry 16

11. Assessment of the MNP@E/EC12 bioconjugate functionality after the covalent bond formation 17

12. Analysis of E-cadherin expression in different cell lines 18

13. MNP cytotoxicity 19

14. Cellular labelling using the bioconjugates MNPs@E/EC12 20

1. E/EC12 cadherin fragments characterization

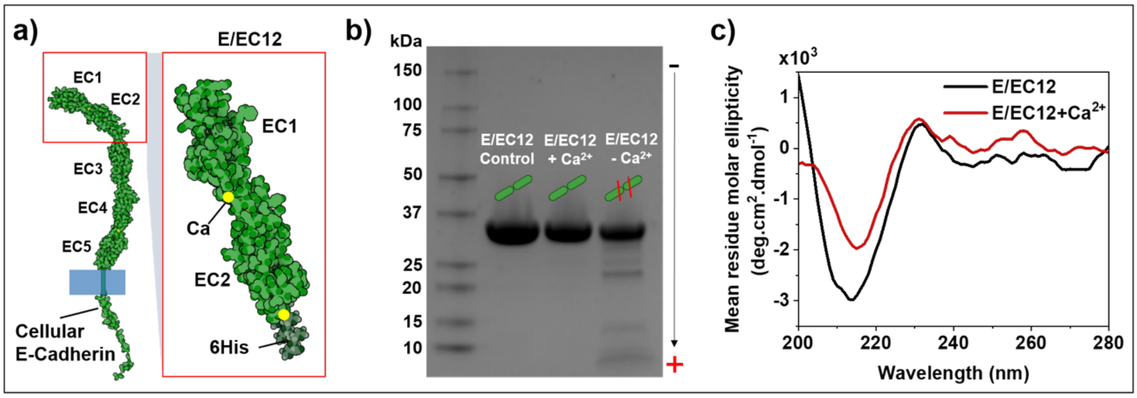

Figure S1: a) Scheme of a cellular E-cadherin and the E/EC12 fragment expressed for the bioconjugation with the MNPs. Protein was rendered using the Protein Imager software.[1] **b)** SDS-PAGE of E/EC12 fragments after 1 h digestion with trypsin in the presence or absence of Ca^{2+} ions; E/EC12 fragments without trypsin digestion were used as a control (lane 1). When treated with trypsin, E/EC12 fragments showed a single band in the presence of calcium (lane 2) but were cleaved in absence of Ca^{2+}(lane 3). **c)** Circular Dichroism spectra of E/EC12 fragments (7 μM) in the absence or the presence of Ca^{2+} ions (0.9 mM).

[1] Tomasello, G.; Armenia, I.; Molla, G. The Protein Imager: A Full-Featured Online Molecular Viewer Interface with Server-Side HQ-Rendering Capabilities. *Bioinformatics* **2020**, *36* (9), 2909–2911. https://doi.org/10.1093/bioinformatics/btaa009.

2. MNP characterization

Transmission electron microscopy (TEM)

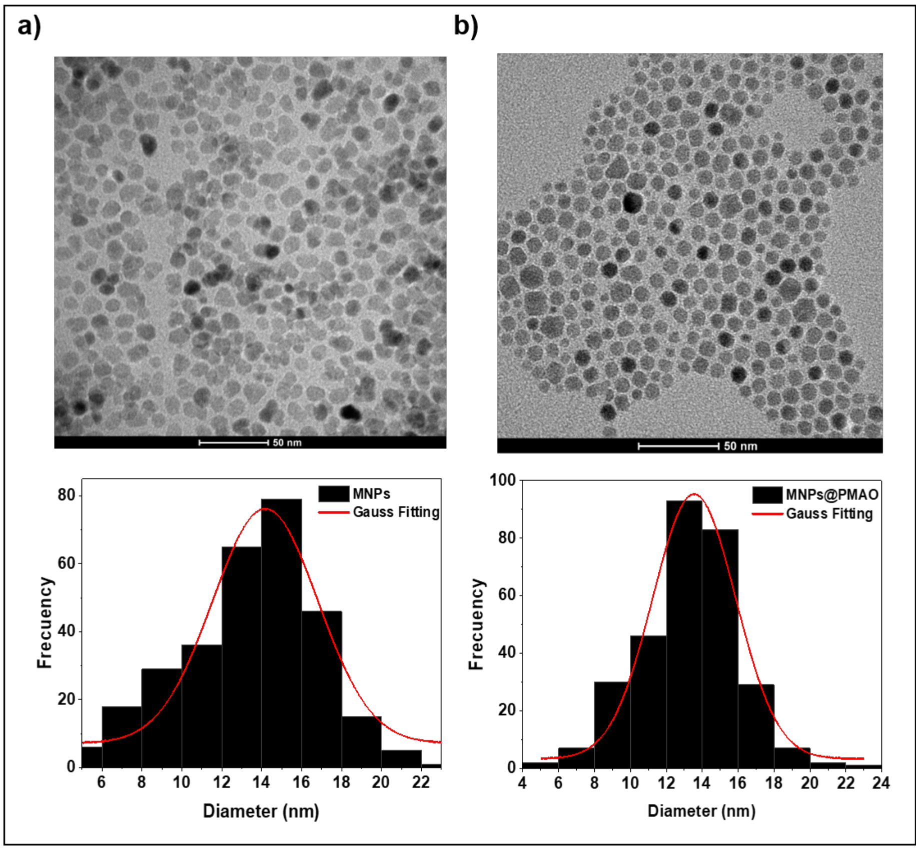

Figure S2: a) TEM micrograph of MNPs in organic media and size distribution (14.2 ± 2.6 nm). b) TEM micrograph of MNPs@PMAO-TAMRA and size distribution (13.6 ± 2.3 nm). Both size distributions were obtained using Fiji software; 300 particles were measured and size was determined using a Gaussian fitting function.

Thermogravimetric analysis of the MNPs

After coating with PMAO, the MNPs were analysed by TGA in order to determine the percentage in weight of the organic shell comprising the oleic acid and the PMAO layer.

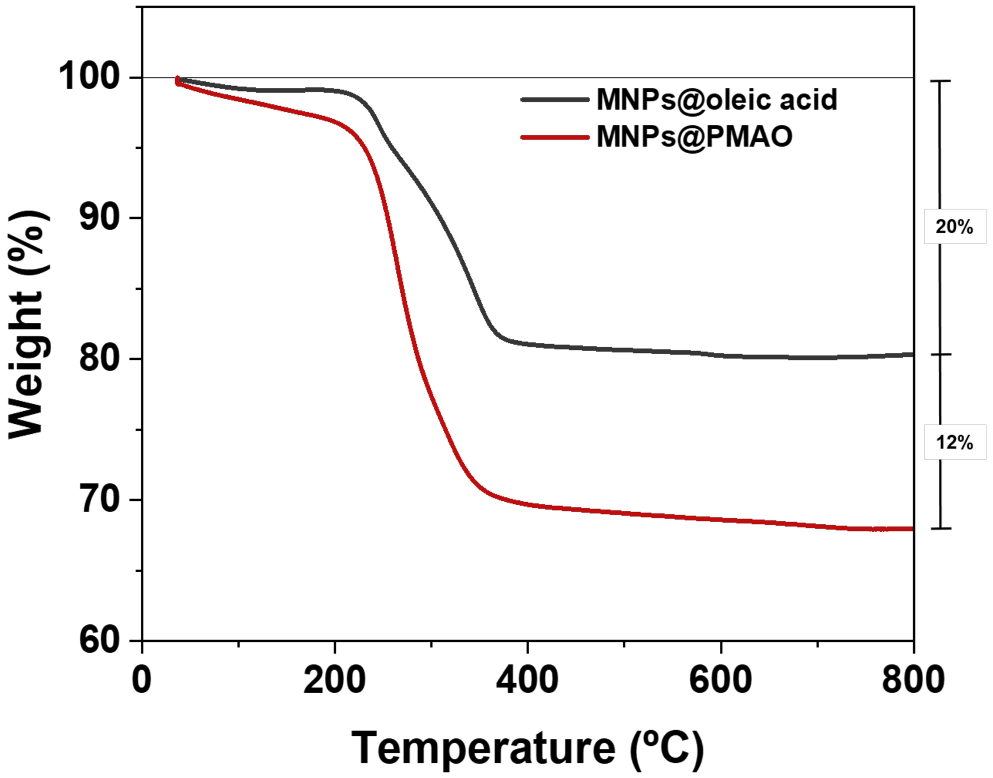

Figure S3: Thermogravimetric analysis (TGA) curves showing the weight loss variations of the organic compounds starting from the MNPs in organic solvent (hexane) (20%), followed by the loss of the PMAO coating (12%).

3. Characterization of MNPs after functionalization with PEG + LysNTA-M^{2+}

After the functionalization by the three different strategies (1, 2A and 2B), the resultant MNPs were analysed by DLS to evaluate differences in size given mainly by the PEG grafting.

Table S1: Hydrodynamic diameter from DLS (by intensity) of MNPs@PMAO-TAMRA functionalized with PEG and LysNTA-M^{2+} by the three strategies.

**Strategy**		**Hydrodynamic diameter (Intensity, nm)**	
**1**	**PEG750**	109 ± 1	
	**PEG5000**	199 ± 24	
**Strategy**		**Step1**	**Step2**
**2A** (1º:PEG; 2º:NTA)	**PEG750**	105 ± 3	103 ± 2
	**PEG5000**	222 ± 30	198 ± 16
**2B** (1º:NTA; 2º:PEG)	**PEG750**	104 ± 2	113 ± 21
	**PEG5000**	104 ± 2	140 ± 28

Table S2: Hydrodynamic diameter from DLS (by number) of MNPs@PMAO-TAMRA functionalized with PEG and LysNTA-M^{2+} by the three strategies.

**Strategy**		**Hydrodynamic diameter (Number, nm)**	
**1**	**PEG750**	32 ± 8	
	**PEG5000**	40 ± 2	
**Strategy**		**Step1**	**Step2**
**2A**	**PEG750**	34 ± 9	25 ± 8
	**PEG5000**	33 ± 5	53 ± 1
**2B**	**PEG750**	27 ± 3	25 ± 5
	**PEG5000**	27 ± 3	47 ± 5

As shown in Table S1, the hydrodynamic diameter of the MNPs grafted with the smaller PEG (750 Da) was similar to MNPs@PMAO-TAMRA, whilst it increased in the MNPs grafted with the larger PEG chains (5000 Da). Those changes after each functionalization step were indicative of a successful functionalization, maintaining a nanometer size with a polydispersity index lower than 0.3.

4. Comparison of the bioconjugation efficiency by using LysNTA complexed with different metallic ions

To test the versatility of our procedure, the bioconjugation was also carried out using Ni^{2+} or Co^{2+} to obtain the LysNTA-M^{2+} complexes (Figure S4a). To further check that both LysNTA complexed with Ni^{2+} and Cu^{2+} behaved similarly in the functionalization process, we carried out the 3 functionalization strategies (1, 2A and 2B) using PEG 5000 and LysNTA-Ni^{2+} instead of LysNTA-Cu^{2+}. Similar to what happened with LysNTA-Cu^{2+} (Figure 2c), all the cadherin binds to the MNPs when using strategy 1 and 2B, while approximately 40% of the protein remains in the supernatant when using strategy 2A (Figure S4b).

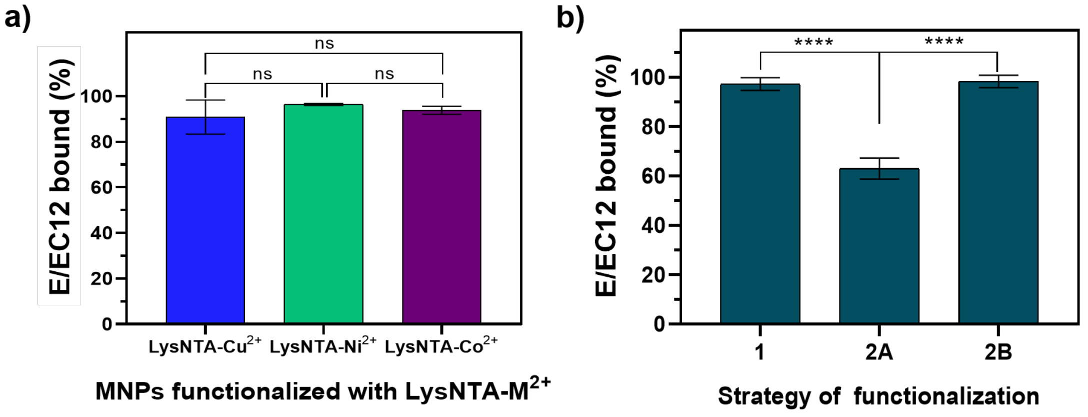

Figure S4: a) Percentage of E/EC12 fragments bioconjugated to MNPs functionalized with LysNTA pre-charged with different divalent cations (Cu^{2+}, Co^{2+}, Ni^{2+}). A ratio of 200 µg E/EC12 fragments/mg Fe was used for the bioconjugation. b) Percentage of E/EC12 fragments bioconjugated to MNPs functionalized with the three functionalization strategies and Lys-NTA complexed with Ni^{2+} (****, $p<0.001$; ns= non-significant; one-way ANOVA, followed by Tukey's multiple comparison test).

5. Characterization of MNPs after functionalization with PEG and different quantities of LysNTA-Ni^{2+}

To modulate the amount of LysNTA-Ni^{2+} complexes functionalized on the MNPs using Strategy 1, we kept the PEG:MNP ratio constant (18 µmols/mg Fe) and varied the amount of LysNTA-Ni^{2+} complexes added during the functionalization (4, 8, 16 and 32 µmols/mg Fe). The resultant MNPs were also characterized by DLS and TGA, in order to confirm the functionalization of PEG molecules.

DLS

Table S3: Hydrodynamic diameter from DLS (by intensity) of MNPs@PMAO-TAMRA functionalized with PEG and four quantities of LysNTA-Ni^{2+} (4, 8, 16 and 32 µmols/mg Fe).

MNPs	D_H by intensity	D_H by number
**@PMAO-TAMRA**	254 ± 56	24 ± 3
**@(4 µmols/mg Fe)**	224 ± 26	33 ± 1
**@(8 µmols/mg Fe)**	186 ± 9	35 ± 2
**@(16 µmols/mg Fe)**	174 ± 25	34 ± 2
**@(32 µmols/mg Fe)**	111 ± 32	33 ± 2

TGA

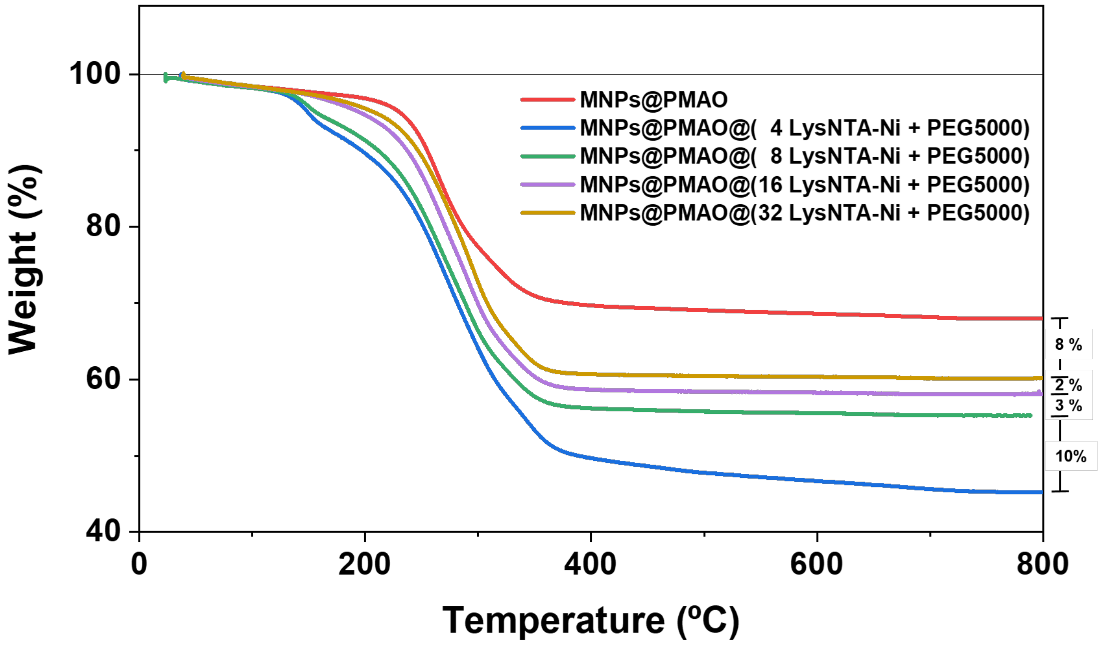

Figure S5: Thermogravimetric analysis (TGA) curves showing the weight loss variations of the organic compounds starting from the PMAO coating and the LysNTA-Ni^{2+} (4, 8, 16 or 32 µmols/mg Fe) + PEG (5000 Da) functionalizations. Here, the lower the quantity of LysNTA-Ni^{2+} added, the higher is the weight loss, suggesting the presence of more PEG molecules.

6- Functionalization with LysNTA or ready-to-use LysNTA-Ni^{2+} complexes

MNPs were functionalized using 64 µmols/mg Fe of LysNTA-Ni^{2+} or LysNTA, while keeping constant the amount of PEG molecules. The samples with LysNTA were then incubated with different excesses of Ni^{2+} (0.64, 64 and 640 µmols of nickel/mg Fe), eliminated its excess by centrifugation and quantified the nickel incorporated by ICP-AES. Only with the amount of 640 µmols of nickel/mg Fe it was possible to obtain a higher incorporation of nickel on the MNPs when compared to the ready-to-use complex (Fig. S6a). However, the colloidal stability of the MNPs was compromised, correlating the loss of stability with the higher amounts of nickel added (64 and 640 µmols of nickel/mg Fe) (Fig. S6b).

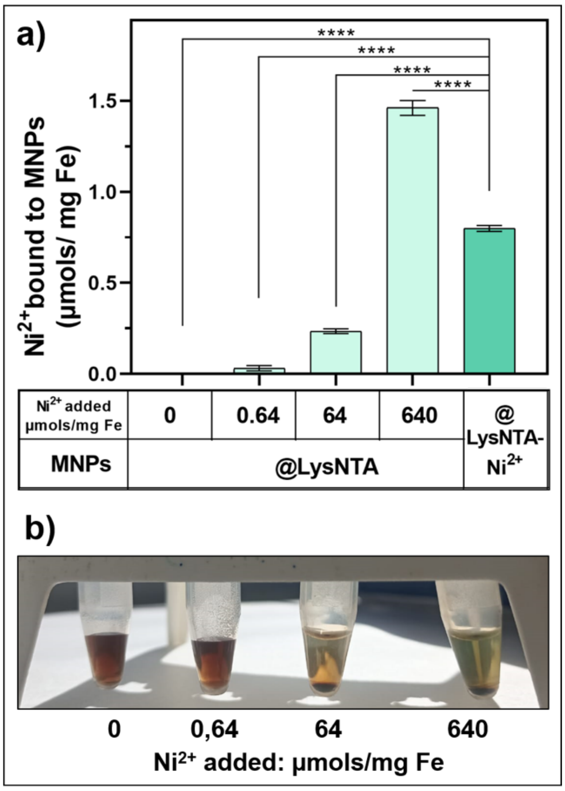

Figure S6. a) Quantity of nickel coordinated on MNPs functionalized with LysNTA- Ni^{2+} or LysNTA (64 µmols/mg Fe). MNPs@LysNTA were then incubated with increasing concentration of Ni^{2+} (0.64, 64 and 640 µmols of nickel/mg Fe). b) Colloidal stability of the MNPs after the addition of free Ni^{2+}.

7. Saturation of the MNP surface

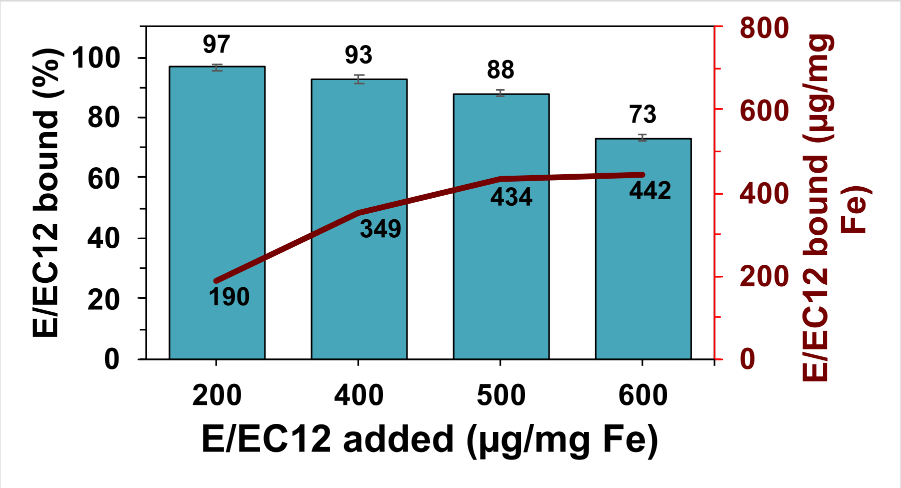

Figure S7. Percentage (%) and amount (µg/mg Fe) of E/EC12 bound to the surface of MNPs functionalized with 32 µmols/mg Fe when different amounts of cadherin fragments were added.

8. Calculation of the number of E/EC12 fragments immobilized/MNP

Calculation S1

8.1. Calculation of the number of MNPs/mg Fe

For simplicity we assumed that the MNPs were composed by magnetite (Fe_3O_4). From the diameter measured by TEM (13.6 nm) we calculated the volume of a single MNP assuming a spherical shape:

$$1.\quad V_{MNP(cm^3)} = \frac{4}{3}.\pi.r^3_{(nm)}.10^{-21}$$

Assuming that the MNP has a similar density to magnetite ($\rho_{Fe_3O_4} = 5,1\frac{g}{cm^3}$) we calculate the mass of a single MNP:

$$2.\quad M_{MNP(g)} = V_{MNP(cm^3)} \cdot \rho_{Fe_3O_4(\frac{g}{cm^3})}$$

Knowing the mass of a MNP, the molecular weight of the magnetite ($MW_{Fe_3O_4} = 231,53\ \frac{g}{mol}$) and the Avogadro's number ($6,022 \cdot 10^{23}\left(\frac{Atoms}{mol}\right)$), we can know the number of iron atoms/MNP and finally, the number of MNPs/mg Fe:

$$3.\quad \frac{No.Atoms_{Fe}}{1\ MNP} = \frac{M_{MNP(g)}}{MW_{Fe_3O_4}} . \frac{3\ mol_{Fe}}{1\ mol_{Fe_3O_4}} . 6,022 \cdot 10^{23}\left(\frac{Atoms}{mol}\right)$$

$$4.\quad \frac{No.MNPs}{mg\ Fe} = \left(\frac{No.Atoms_{Fe}}{1\ MNP} . \frac{1g_{Fe}}{No.Atoms_{Fe}}\right)^{-1} . 10^{-3}$$

8.2. Calculation of the number of E/EC12 fragments immobilized per MNP

First, the concentration of E/EC12 fragments functionalized $C_{functionalized}$ was obtained by difference between the stock concentration and the quantity of non-bound protein ($C_{supernatant}$):

$$5.\quad C_{functionalized}\left(\frac{\mu g}{mL}\right) = C_{stock} - C_{supernatant}\left(\frac{\mu g}{mL}\right)$$

Then, with the $C_{functionalized}$, the total mass of protein functionalized was calculated having in mind the final volume of medium used during the reaction consisting on the sum of the MNPs volume (V_{NPs}) and the protein volume ($V_{Protein}$):

$$6.\quad \mu g_{functionalized} = C_{functionalized}\left(\frac{\mu g}{mL}\right) \cdot (V_{NPs} + V_{Protein})(mL)$$

The number of E/EC12 fragments/0.1 mg Fe is calculated with the molecular weight of the E/EC12 fragments ($MW_{E-cad\,frag.} = 25205\ \frac{g}{mol}$) and the Avogadro's number:

$$7.\quad \frac{No\ of\ Fragments}{0.1\ mg\ Fe} = \mu g_{functionalized} \cdot \frac{1}{MW_{E-cad\,frag}} \cdot \frac{6.022 \cdot 10^{23} molecules}{1\ mol} . 10^{-6}$$

Finally, once obtained the $\frac{No\ of\ Fragments}{0.1\ mg\ Fe}$, the $\frac{No\ of\ Fragments}{NP}$ is calculated using the ratio obtained above for the number of MNPs/mg Fe as follows:

$$8.\quad \frac{No\ of\ Fragments}{NP} = \frac{No\ of\ Fragments}{0.1\ mg\ Fe} \cdot \frac{1\ mg\ Fe}{2.91 * 10^{14} NPs}$$

$$9.\quad \frac{No\ of\ Fragments}{nm^2} = \frac{No\ of\ Fragments}{NP} \cdot \frac{1\ NP}{\pi * D^2}$$

9. Covalent coupling

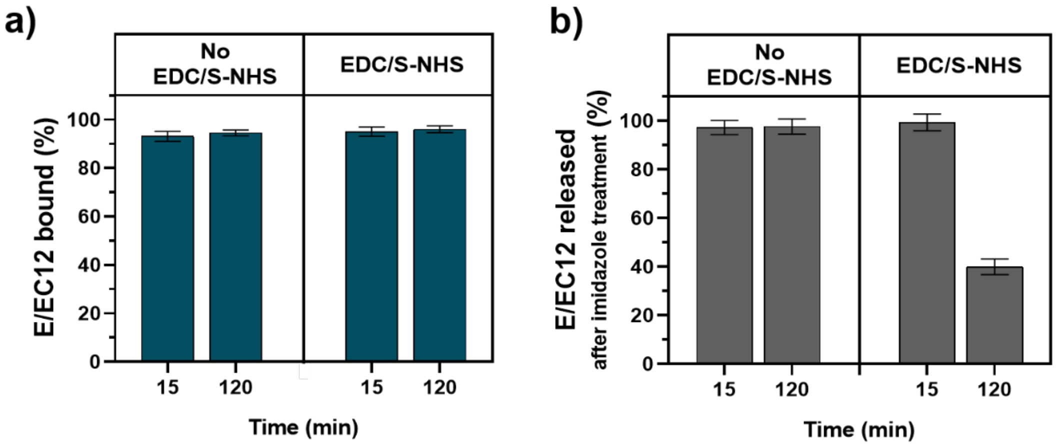

Figure S8. Covalent attachment using strategy i). a) Percentage (%) of E/EC12 bound to the surface of MNPs activated or not with EDC/S-NHS. b) Percentage (%) of E/EC12 released from the surface of MNPs (activated or not with EDC/S-NHS) when imidazole 0.5 M was added.

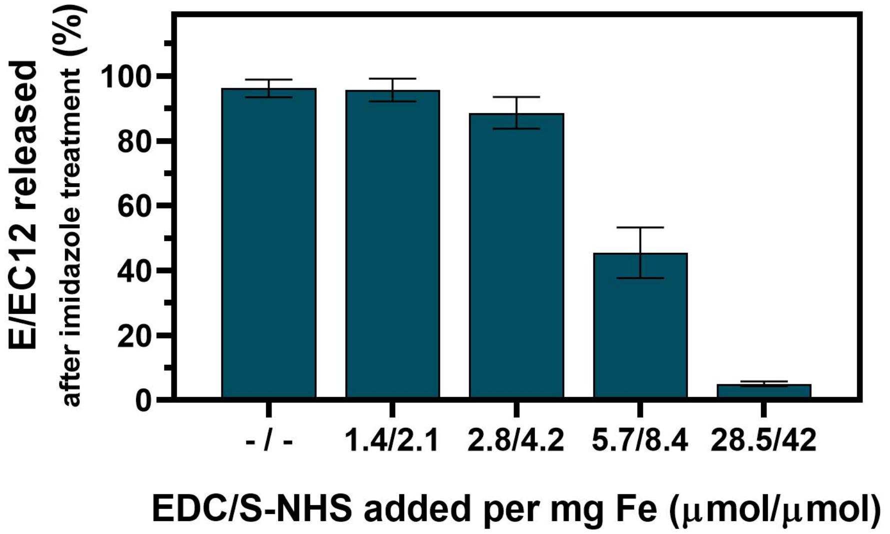

Figure S9. Covalent attachment using strategy ii). Percentage (%) of E/EC12 released from the surface of MNPs when imidazole 0.5 M was added.

10. Assessment of the MNP@E/EC12 bioconjugate functionality by magnetic resonance relaxometry

When MNP@E/EC12 were incubated in the absence of calcium, their T_2 values remained constant during the whole experiment (12 hours, Fig. S10). However, in the presence of calcium, the T_2 increased after 12 hours of incubation due to the aggregation and precipitation of the MNPs (less MNPs remained in solution, see Fig. 6c). However, upon the addition of EDTA the aggregation was reversed, resulting again in a decrease of the T_2 value (Fig. S10).

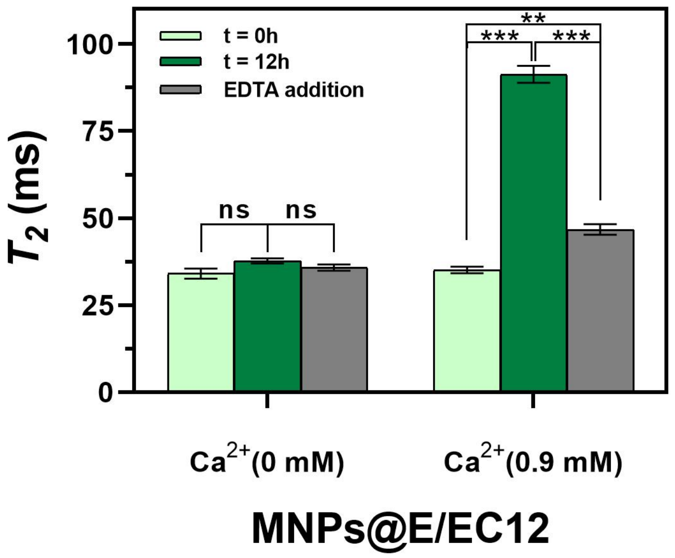

Figure S10: T_2 (ms) variation after bioconjugates incubation in dPBS (Ca^{2+} 0.9 mM) or PBS (Ca^{2+} 0 mM) during 12 hours and subsequent EDTA addition (5 mM) during 1 hour. Black asterisks indicate statistical differences (ns= non-significant; $^{**}p < 0.01$; $^{***}p < 0.001$; one-way ANOVA, followed by Tukey's multiple comparison test.

11. Assessment of the MNP@E/EC12 bioconjugate functionality after the covalent bond formation

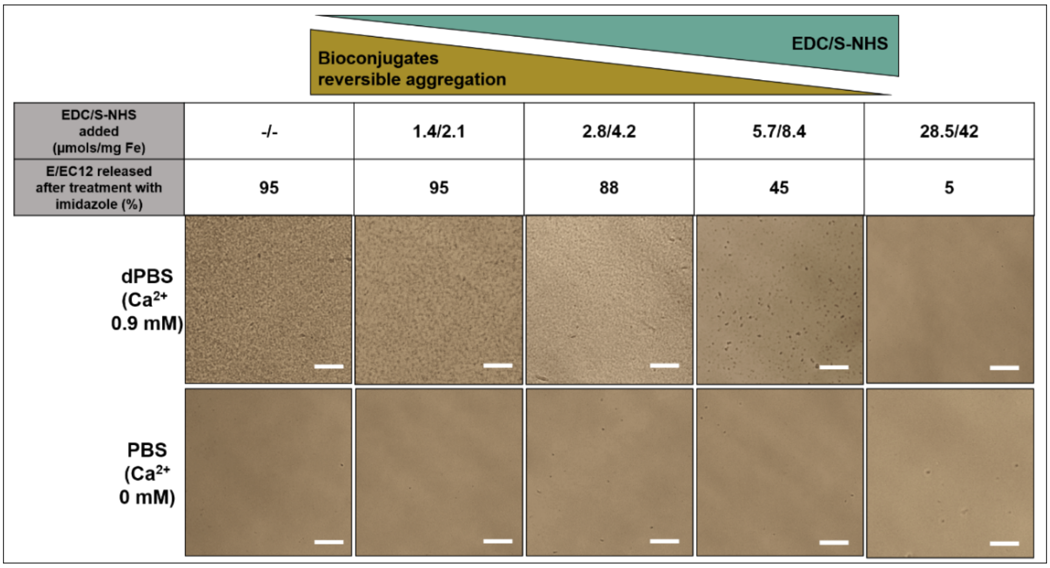

Figure S11. Analysis of the functionality of the MNP@E/EC12 bioconjugates in which the protein was covalently bound following strategy ii). Only MNPs where low quantities of EDC/S-NHS were added remained functional (aggregate formation in the presence of Ca^{2+}). Scale bar: 50 μm.

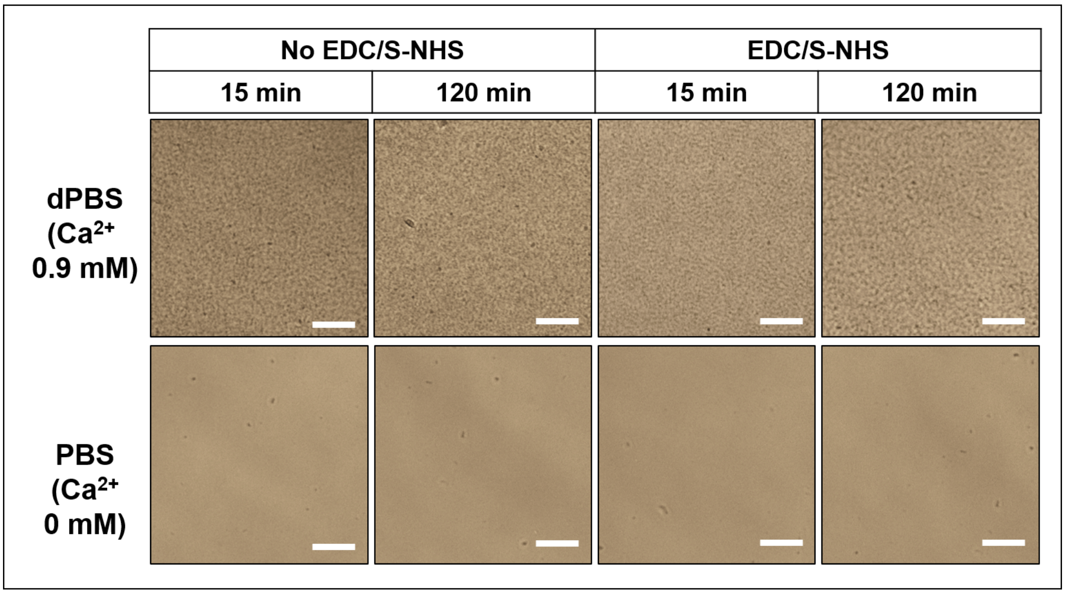

Figure S12. Analysis of the functionality of the MNP@E/EC12 bioconjugates in which the protein was covalently bound following strategy i). All MNPs were able to form aggregates in the presence of Ca^{2+}, independently of their activation with EDC/S-NHS. Scale bar: 50 μm.

12. Analysis of E-cadherin expression in different cell lines

The presence of E-cadherin on the cell membrane of both cell lines (HC11 and NIH-3T3) was evaluated by flow cytometry. In control samples, only secondary antibody was added to evaluate non-specific binding. As shown below, a clear shift of the peak was only shown for HC11 cells, confirming the presence of E-cadherin on their membrane.

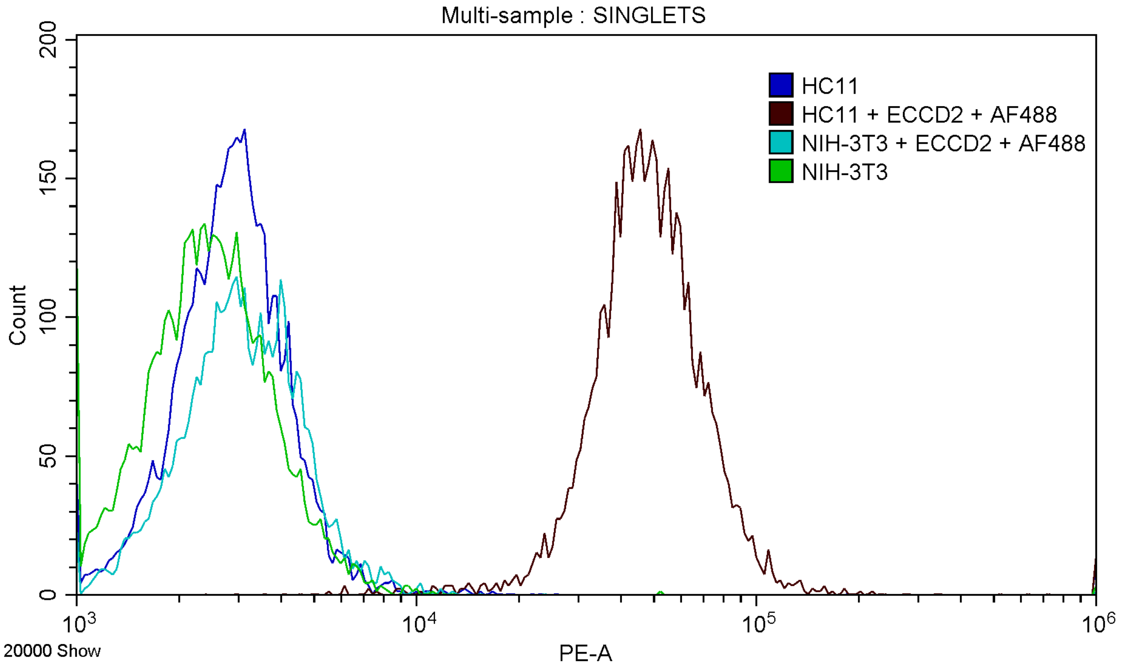

Figure S13: Flow cytometry of E-cadherin expression by indirect immunostaining with anti E-cadherin (ECCD2) primary antibody and secondary anti-rat antibody modified with Alexa Fluor 488.

13. MNP cytotoxicity

HC11 cells were seeded (3.2×10^4 cells/well) in a standard 96-well plate (200 µL/well) and incubated for 24 h in cell culture conditions. Cells were incubated with five different concentrations (25, 50, 100, 150 and 250 µg Fe/mL) of the MNPs@LysNTA-Ni^{2+} (32 µmols/mg Fe) in RPMI, with three replicates per concentration and a negative control (non- treated cells). After 24 hours of incubation, the wells were washed twice with RPMI to remove the nanoparticles and fresh medium containing MTT dye solution (0.25 mg/mL in RPMI) was added to each well. 1 hour and half later the supernatant was removed and the formazan crystals were solubilized with 100 µL of dimethyl sulfoxide (DMSO) for 15 minutes. After mixing, the optical density at 570 nm was recorded using a Thermo Scientific Multiskan GO™ microplate reader. The relative cell viability (%) related to control cells without treatment was calculated using the percentage ratio between absorbance of the sample and the absorbance of the control.

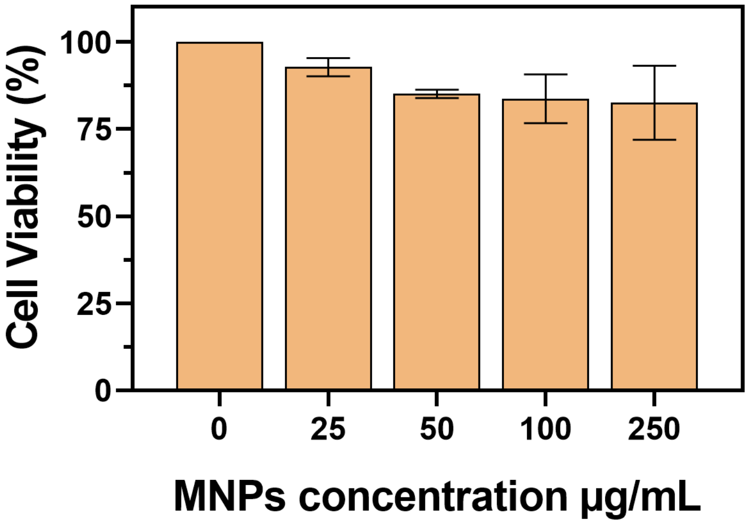

Figure S14: Cytotoxicity of MNPs@LysNTA-Ni^{2+} (32 µmols/mg Fe) in HC11 cells after 24 hours of incubation. Experiments were performed in triplicate, and data are represented as the mean value ± the standard deviation.

14. Cellular labelling using the bioconjugates MNPs@E/EC12

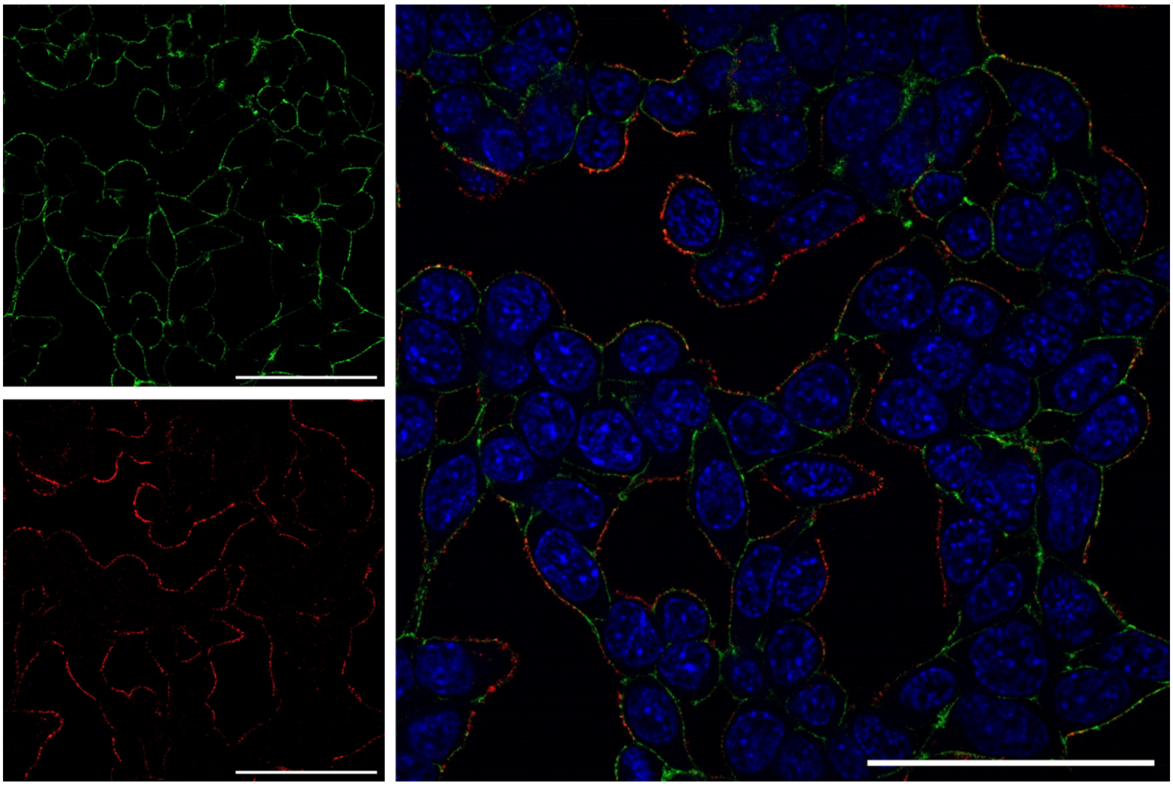

Figure S15: Confocal microscopy image of mouse epithelial cells (HC11) incubated with MNPs@E/EC12. Cellular E-cadherins were immunostained and are shown in green (AF488); MNPs containing TAMRA are in red; nuclei are in blue; scale bars: 50 µm.
